# *HLA* genotype testing for carbamazepine, oxcarbazepine and eslicarbazepine: A guideline developed by the UK Centre of Excellence in Regulatory Science and Innovation in Pharmacogenomics (CERSI‐PGx)

**DOI:** 10.1002/bcp.70559

**Published:** 2026-04-19

**Authors:** Lucy Galloway, Cinzia Dello Russo, Nicholas Bass, Elvira Bramon, Helen Cross, Natalie Curley, Sarah Curran, Helen Davies, Jana De Villiers, William Evans, Bernhard Frank, Alice Groves, Judith Hayward, Jon Higham, Dyfrig A. Hughes, Shwe Sin Kyaw, Anthony G. Marson, Ailsa McLellan, Seth Mensah, Francis O'Neill, Jane Sargison, Sanjay M. Sisodiya, Jill Swan, Joanna M. Zakrewska, Munir Pirmohamed

**Affiliations:** ^1^ NHS South East Genomic Medicine Service St George's University Hospitals NHS Foundation Trust London UK; ^2^ Faculty of Life Sciences & Medicine King's College London London UK; ^3^ Department of Pharmacology and Therapeutics, Institute of Systems Molecular and Integrative Biology University of Liverpool Liverpool UK; ^4^ Department of Translational Medicine and Surgery, Section of Pharmacology Università Cattolica del Sacro Cuore—Fondazione Policlinico Universitario A. Gemelli, IRCCS Rome Italy; ^5^ Division of Psychiatry University College London London UK; ^6^ North London NHS Foundation Trust London UK; ^7^ University College London Great Ormond Street Institute of Child Health London UK; ^8^ Great Ormond Street Hospital for Children NHS Foundation Trust London UK; ^9^ NHS South West London Integrated Care Board London UK; ^10^ South London and Maudsley NHS Foundation Trust London UK; ^11^ Cwm Taf Morgannwg University Health Board Abercynon UK; ^12^ The State Hospitals Boards for Scotland Lanark UK; ^13^ Yorkshire Regional Genetics Service Leeds UK; ^14^ Gibson Lane Practice Leeds UK; ^15^ School of Medicine University of Nottingham Nottingham UK; ^16^ The Walton Centre NHS Foundation Trust Liverpool UK; ^17^ Urgent Care Division Aneurin Bevan University Health Board Newport UK; ^18^ National Genomics Education Programme, NHS England London UK; ^19^ NHS North West Genomic Medicine Service Manchester UK; ^20^ Shipley Medical Practice Affinity Care Shipley UK; ^21^ Birmingham Community Healthcare NHS Foundation Trust and Birmingham Dental Hospital Birmingham UK; ^22^ Centre for Health Economics and Medicines Evaluation North Wales Medical School Bangor University Bangor UK; ^23^ Royal Hospital for Children and Young People Edinburgh UK; ^24^ Cardiff and Vale University Health Board Cardiff UK; ^25^ School of Dentistry, Institute of Life Course and Medical Sciences University of Liverpool Liverpool UK; ^26^ Faculty of Science and Engineering Manchester Metropolitan University Manchester UK; ^27^ Department of Epilepsy, UCL Queen Square Institute of Neurology University College London London UK; ^28^ National Hospital for Neurology and Neurosurgery and Chalfont Centre for Epilepsy University College London Hospitals NHS Foundation Trust London UK; ^29^ NHS Ayrshire and Arran Ayr UK; ^30^ Royal National ENT & Eastman Dental Hospitals University College London Hospitals NHS Foundation Trust London UK; ^31^ The Wolfson Centre for Personalised Medicine, Centre for Drug Safety Science University of Liverpool Liverpool UK

**Keywords:** carbamazepine, eslicarbazepine, HLA‐A, HLA‐B, hypersensitivity, oxcarbazepine, pharmacogenomics

## Abstract

Carbamazepine is licensed in the United Kingdom for the treatment of epilepsy, bipolar disorder and trigeminal neuralgia. The related compounds oxcarbazepine and eslicarbazepine are licensed for the treatment of epilepsy. These drugs can cause immune‐mediated hypersensitivity reactions, which typically affect the skin, and can be of variable severity. The liver and other organ systems can also be affected. The *HLA* alleles, HLA‐B*15:02, HLA‐B*15:11 and HLA‐A*31:01, are known predisposing factors for these hypersensitivity reactions. Any treatment‐naïve patient, regardless of ancestry or indication for treatment, who is about to be prescribed carbamazepine, oxcarbazepine or eslicarbazepine, or has been on these drugs for less than 3 months, should undergo pharmacogenetic testing to identify all clinically relevant *HLA* alleles to reduce the risk of hypersensitivity reactions. Carbamazepine, oxcarbazepine and eslicarbazepine should be avoided in HLA‐B*15:02‐positive patients. These drugs should also be avoided in patients positive for HLA‐A*31:01 or HLA‐B*15:11 if an alternative is possible. Where it is not possible to use an alternative, treatment should only be commenced after careful consideration of the benefits and risks, with increased monitoring and advising patients on appropriate action to take if a skin rash occurs. Our guideline is compatible with other international pharmacogenetics prescribing guidelines. This guideline is grounded in the latest evidence but cannot account for all individual factors relevant to patient care. Therefore, prescribers must conduct a thorough assessment of each patient's risk–benefit profile, ensuring that therapy is optimised to maximise benefits whilst minimising potential harms.

## 1 BACKGROUND AND OVERVIEW

Carbamazepine is an antiseizure medication with an aromatic ring chemical structure related to tricyclic antidepressants. The drug reduces the propagation of abnormal action potentials in the brain by producing a frequency‐ and voltage‐dependent block of sodium channels, thereby reducing the generation of repetitive action potentials in epileptic foci.^1^ Therapeutic drug monitoring is available to maintain therapeutic serum concentrations within the range 4–12 μg/mL for the treatment of epilepsy^2^ but is variably utilised. Dose‐dependent (and serum concentration–dependent) adverse reactions include diplopia, drowsiness, nausea and sedation.

Type B adverse reactions to carbamazepine do not have a simple linear relationship with either dose or serum concentration and have a complex underlying immune pathophysiology.^1^ Such reactions include different cutaneous adverse reactions, such as maculopapular exanthema (MPE), drug hypersensitivity syndrome (HSS) also called drug rash with eosinophilia and systemic symptoms (DRESS), Stevens–Johnson syndrome (SJS) and toxic epidermal necrolysis (TEN).^3^ SJS is characterised by mucosal involvement and skin/epidermal detachment affecting up to 10% of body surface area, whereas TEN usually involves >30% of the body surface area. Overlap syndrome is characterised by patients with 10%–30% body surface area detachment.^3^, ^4^ Mortality rates are up to 5% for SJS and >30% for TEN. In addition to the cutaneous hypersensitivity reactions, carbamazepine can also affect other organs including the liver (hepatotoxicity), kidneys (nephritis) and lung (pneumonitis), either in isolation or in combination with cutaneous involvement. The incidence of SJS/TEN in patients treated with carbamazepine has been reported to be 0.25% (one in 400) in Han Chinese and 0.005% (one in 20 000) in Europeans, whereas the incidence of DRESS is estimated around 0.05% (one in 2000) in both Chinese and Europeans.^5^

Oxcarbazepine is a structural analogue of carbamazepine, developed to minimise adverse effects related to carbamazepine and its metabolites.^6^ The inhibition of sodium channels by oxcarbazepine occurs at lower concentrations in vitro in comparison to carbamazepine, and the two drugs have different inhibitory actions on calcium channels expressed in the central nervous system.^7^ There are also important differences in the metabolism of the two drugs, with cytochrome P450 enzymes being more important for the metabolism of carbamazepine compared with oxcarbazepine.^6^ Oxcarbazepine has been reported to cause cutaneous eruptions less frequently than carbamazepine,^7^ but is not devoid of causing hypersensitivity reactions including more serious reactions such as SJS/TEN. The incidences of oxcarbazepine‐induced SJS/TEN and DRESS are as follows: <0.01% (less than one in 10 000) and between 0.01% and 0.1% (between one in 10 000 and one in 1000), respectively.^5^

Oxcarbazepine is a prodrug that is rapidly metabolised to the active metabolite 10‐monohydroxy oxcarbazepine, also called licarbazepine. The active enantiomer S‐licarbazepine (eslicarbazepine) has been developed as a drug in the form of eslicarbazepine acetate, for use as both monotherapy and adjunctive therapy for focal seizures.^8^ Eslicarbazepine shows reduced pharmacokinetic and pharmacodynamic interactions and it can be administered once daily.^9^ However, eslicarbazepine, like carbamazepine and oxcarbazepine, can also cause immune‐mediated hypersensitivity reactions.

Different *HLA* genomic biomarkers have been identified as predisposing factors (covered in more detail in Section 3) for these immune‐mediated hypersensitivity reactions (see Table 1). Their frequency varies amongst populations and is also variably reported in pharmacogenetic guidelines and databases.^5^, ^10^ For example, the allelic frequency of HLA‐B*15:02 is highest in Southeast Asian (0%–36%) and South Asian (0%–14%) populations (Table 1). However, this allele is not found at such high frequencies in all Asian subpopulations, for example, the frequency is low in Japanese (<1%).^5^ It is also rare in African, Latino or European ancestry groups.^10^ The allelic frequency of HLA‐A*31:01 ranges between 0% and 19% in Asian, European and Latino ancestry groups, whereas it is rare in African populations (Table 1). The HLA‐B*15:11 allele has an increased frequency in certain Asian populations including up to 12.9% in a Chinese population from Beijing.^11^

**TABLE 1:** Frequencies of *HLA* alleles in different biogeographical groups.

*HLA* alleles	African (sub‐Saharan)	African American/Afro‐Caribbean	Asian (Southeast)	Asian (South)	European	Latino	Oceanian
HLA‐B*15:02	0%–1.6%	0%–0.1%	0%–35.8%	0%–14.2%	0%–1%	0%–0.2%	1%–22%
HLA‐B*15:11	0%	0%	0%–12.9%	0%–0.1%	0%–0.3%	0%	0%–0.5%
HLA‐A*31:01	0%–3.5%	0.1%–3.8%	0.4%–2.8%	0%–19%	0%–6.8%	2.7%–5.3%	0%–10.5%

*Note*: The allelic frequencies have been taken from the HLA Allele Frequency Net Database.^11^

The *HLA* allele frequencies may be useful for a broad evaluation of ‘at risk’ populations, although they cannot replace genotyping on an individual basis. Patients may not always be aware of or disclose biogeographical ancestry. Clinicians should be aware that published allelic frequencies may vary across subpopulations, studies and databases.^5^, ^10^

## 2 LICENSED INDICATIONS

**Table:** 

**Currently licensed indications for carbamazepine,** ^12^ **oxcarbazepine** ^13^ **and eslicarbazepine** ^14^ **in the United Kingdom**
**Carbamazepine is indicated in:** **Epileps*y*** *—generalised tonic–clonic and partial seizures in adults and children*. **Trigeminal neuralgia** *—paroxysmal pain of trigeminal neuralgia in adults*. **Manic‐depressive psychosis** *—prophylaxis of manic‐depressive psychosis in adults unresponsive to lithium therapy*. **Oxcarbazepine is indicated in:** **Epilepsy** *—partial seizures with or without secondary generalised tonic–clonic seizures in adults and children aged above 6 years*. **Eslicarbazepine is indicated in:** **Epilepsy** *—partial‐onset seizures, with or without secondary generalisation in adults and children aged above 6 years*.

***Disclaimer:*** *The exact wording for the currently licensed indications has been extracted from the summaries of product characteristics (SmPCs) of Tegretol®, Trileptal® and Zebinix® approved by the Medicines Healthcare products Regulatory Agency (MHRA), date of revision of the text: Tegretol® 22/12/2023, Trileptal® 28/07/2025 and Zebinix® 01/01/2021. Licensed indications may vary between products and formulations, for example, carbamazepine suppositories are licensed in epilepsy only. In the Tegretol® SmPC focal seizures are reported as partial seizures and bipolar disorder is reported as manic‐depressive psychosis. Likewise, in both Trileptal® and Zebinix® SmPCs focal seizures are reported as partial seizure*s.

These drugs can be prescribed by any prescriber if it is within their competence. In practice however, for epilepsy or bipolar disorder, these drugs are largely prescribed by neurology and psychiatry. For trigeminal neuralgia, the prescriber pool for carbamazepine is larger and includes (but not limited to) primary care, dentistry, neurology, neurosurgery, pain medicine, oral medicine and oral surgery. It is important to note that routine prescribing of carbamazepine, oxcarbazepine and eslicarbazepine has changed over time because of various factors including (a) the introduction of alternative drugs and (b) changes in clinical practice. Recent guidance from the MHRA restricting the use of valproate‐containing medicines for epilepsy and bipolar disorder due to reproductive risks may increase the use of carbamazepine, oxcarbazepine and eslicarbazepine.^15^

## 3 EVIDENCE OVERVIEW

Seminal studies in the Han Chinese population have consistently reported a strong association between the HLA‐B*15:02 allele and carbamazepine‐induced SJS/TEN.^3^, ^16^, ^17^ The association has also been observed in Thai, Malay and Indian populations.^18^, ^19^, ^20^, ^21^, ^22^ Many systematic reviews and meta‐analyses have been undertaken in Southeast Asian populations, and all of them have consistently shown a strong association between carbamazepine‐induced SJS/TEN and HLA‐B*15:02 with an odds ratio (OR) of 48.5 (95% Confidence Interval [CI], 23.7–99.5) reported in a more recent paper.^23^ It is important to note that the association between HLA‐B*15:02 and carbamazepine has only been with SJS and TEN and not with other hypersensitivity phenotypes. Moreover, the strength of the association has been confirmed in clinical indications other than epilepsy. For example, a study in Thai patients treated with carbamazepine for neuropathic pain showed that the allele was found in 32/34 SJS/TEN patients, compared with 7/40 control patients (OR 75.4; 95% CI, 13.0–718.9).^19^ The study population included patients with trigeminal neuralgia, 32.3% amongst the cases and 37.5% amongst the controls.

The association with HLA‐B*15:02 has also been identified in the paediatric population.^24^, ^25^ A study in Singapore in 32 paediatric cases, five with SJS/TEN (two Chinese and three Malay), six with HSS (five Chinese and one Indian), 11 with minor drug reactions (nine Chinese and two Malay) and 10 controls (seven Chinese, two Malay and one Indian) showed that the HLA‐B*15:02 was positive in 100% of patients with SJS/TEN (OR 27.20; 95% CI, 2.67–∞) but not with HSS (OR 1.67; 95% CI, 0–65) or minor drug reactions (OR 0.90; 95% CI, 0.01–78.41).^25^

Studies in Caucasian^26^, ^27^ and Japanese^28^ populations have failed to demonstrate an association between HLA‐B*15:02 and carbamazepine‐induced SJS/TEN. This is because of the low frequency of the HLA‐B*15:02 allele in these populations (Table 1). However, in the rare individuals with European ancestry who are positive for HLA‐B*15:02, it can be assumed that the risk of SJS‐TEN will be equivalent to that seen in Southeast Asian populations.

The clinical utility of pharmacogenetic testing for HLA‐B*15:02 has been demonstrated by Chen and collaborators.^29^ In a prospective cohort study of 4855 Han Chinese patients, pre‐emptive genotyping for HLA‐B*15:02 identified 372 patients (7.7%) positive for HLA‐B*15:02 who were then excluded from receiving carbamazepine. None of the patients treated with carbamazepine were diagnosed with SJS/TEN.^29^ Based on the incidence of SJS/TEN between 2002 and 2004—a mean of 0.23% (one in 435)—the authors calculated that approximately 10 cases of SJS or TEN would be expected in the 4120 subjects treated with carbamazepine. Notably, the majority of patients enrolled in the study (54.1%) were diagnosed with trigeminal neuralgia, 14.2% with epilepsy and 2.8% with bipolar disorder, highlighting the relevance of the gene–drug association and the utility of pharmacogenetic testing across the different indications. A 2012 systematic review of 23 studies investigating the association between carbamazepine‐induced cutaneous adverse drug reactions and HLA genotype estimated that the number needed to test (NNT) to prevent one case of carbamazepine‐induced SJS/TEN in Asian patients has been estimated to be 461, assuming that the incidence of carbamazepine‐induced SJS/TEN is 23 per 10 000.^21^

An association between oxcarbazepine‐induced serious cutaneous adverse reactions (including SJS/TEN) and HLA‐B*15:02 has also been demonstrated in a meta‐analysis of three studies (OR 18.13; 95% CI, 6.77–48.56).^30^ Molecular modelling studies have shown that oxcarbazepine binds to the HLA‐B*15:02 allele in a similar way to carbamazepine providing mechanistic evidence for the role of this *HLA* allele.^31^ The evidence for an association between eslicarbazepine‐induced SJS/TEN toxicity and HLA B*15:02 is not robust^32^, which may reflect its very low usage. However, given that it is structurally and functionally closely related to carbamazepine and is the active metabolite of oxcarbazepine, it is likely that there is an association between HLA‐B*15:02 and eslicarbazepine‐induced SJS/TEN.

In a European ancestry population, an association was demonstrated between HLA‐A*31:01 and carbamazepine‐induced HSS (DRESS)^33^ with an OR of 12.41 (95% CI, 1.27–121.03). The association was also shown in patients with MPE (OR 8.33; 95% CI, 3.59–19.36) and SJS‐TEN (OR 25.93; 95% CI, 4.93–116.18). The same association was also shown in Japanese patients^34^ with MPE, DRESS and SJS‐TEN (OR 10.8; 95% CI, 5.9–19.6). Since then, the association between HLA‐A*31:01 and various adverse cutaneous phenotypes caused by carbamazepine has been demonstrated in many other populations including those from Southeast Asia. There are seven meta‐analyses confirming the strength of this association.^5^ The association seems strongest for DRESS and MPE but less so for SJS‐TEN.^35^, ^36^ Carbamazepine‐induced liver injury has also been associated with HLA‐A*31:01.^37^ The association with HLA‐A*31:01 and carbamazepine‐induced DRESS (OR 26.4) and MPE (OR 8.6), but not with SJS‐TEN, has also been demonstrated in a paediatric population.^38^ The association between oxcarbazepine‐ and eslicarbazepine‐induced cutaneous adverse reactions and HLA‐A*31:01 is not as robust as with carbamazepine, largely having been described in case reports.^32^, ^39^, ^40^

The clinical utility of HLA‐A*31:01 genotyping to prevent carbamazepine‐induced hypersensitivity reactions has been demonstrated in a prospective cohort study from Japan.^41^ Out of 1130 patients included in the study, 198 (17.5%) were positive for HLA‐A*31:01 and were therefore prescribed drugs other than carbamazepine. Compared with historical controls, the incidence of carbamazepine‐induced cutaneous adverse reactions was reduced by 40% (OR 0.6; 95% CI, 0.36–1.00) using BioBank Japan data for comparison or by 61% (OR 0.39; 95% CI, 0.26–0.59) when compared with the Japan Medical Centre claims database. A UK analysis indicated that in northern Europeans, for HLA‐A*31:01, the NNT was 125 to prevent one case of carbamazepine‐induced cutaneous adverse reaction or 3667 to prevent one case of carbamazepine‐induced HSS/SJS/TEN.^42^

The HLA‐B*15:11 allele has also been identified as another potential pharmacogenetic predictor for carbamazepine induced severe cutaneous reactions. HLA‐B*15:11 belongs to the HLA‐B75 serotype along with HLA‐B*15:02 and HLA‐B*15:21, and the shared structural features likely contribute to similar immunologic predisposition to SJS‐TEN through drug–antigen presentation to T‐cells.^43^ A meta‐analysis showed a significant association of HLA‐B*15:11 with carbamazepine‐induced cutaneous adverse drug reactions (OR 6.08; 95% CI, 2.28–16.23), independent of HLA‐B*15:02.^44^ More recently, the association between HLA‐B*15:11 and an increased risk of SJS/TEN due to carbamazepine was confirmed in a cohort of Japanese subjects (OR 15.8; 95% CI, 6.6–37.7) and validated through a meta‐analysis using results from previous studies carried out in Korea, Southern China and Thailand (OR 16.6; 95% CI, 8.1–34.1).^35^

There is evidence of an association between HLA‐B*15:02 and other aromatic antiepileptic agents including phenytoin, fosphenytoin and lamotrigine. There is limited evidence of an association between HLA‐B*15:02 and phenobarbital. Although recommendations for *HLA* pharmacogenetic testing for other aromatic antiepileptic agents is beyond the scope of this guideline, this may have implications for choosing alternatives to carbamazepine, oxcarbazepine or eslicarbazepine in patients who are positive for HLA‐B*15:02. A summary of the evidence of the association between HLA‐B*15:02 and other aromatic antiepileptic agents is included in Section 11.

## 4 RECOMMENDED INDICATIONS FOR PHARMACOGENETIC TESTING

### 4.1 Patients who have no or less than 3 months of exposure to carbamazepine, oxcarbazepine or eslicarbazepine

Any treatment naïve patient, regardless of ancestry or indication for treatment, who is about to be prescribed carbamazepine, oxcarbazepine or eslicarbazepine should undergo pharmacogenetic testing to identify all clinically relevant *HLA* alleles to reduce the risk of immune‐mediated hypersensitivity reactions. Patients who have previously taken carbamazepine, oxcarbazepine or eslicarbazepine for less than 3 months should also undergo testing.

Pharmacogenetic testing may not be required if pharmacogenetic information obtained from an accredited laboratory (see Section 6) is already available in their medical record.

The availability of pharmacogenetic testing for HLA‐B*15:02, HLA‐B*15:11 and HLA‐A*31:01 may vary in time, indication and in geography. In the absence of available relevant pharmacogenetic information or testing, the current best practice clinical guidelines should be followed. We recommend exercising caution if presented with pharmacogenetic test results from direct‐to‐consumer or commercial providers that lack proper accreditation and robust external quality assurance procedures. Healthcare professionals are advised not to take results from nonaccredited laboratories at face value.^45^

### 4.2 Patients who have more than 3 months of exposure to carbamazepine, oxcarbazepine or eslicarbazepine

If a patient has taken carbamazepine, oxcarbazepine or eslicarbazepine previously for more than 3 months, it is highly unlikely that a severe cutaneous reaction will occur after that time and pharmacogenetic testing will be less helpful for treatment‐experienced patients compared to treatment‐naïve patients.^1^

## 5 INTEGRATING PHARMACOGENETIC TESTING INTO EXISTING CLINICAL PATHWAYS

### 5.1 Epilepsy

For treatment of epilepsy in adults, young people and children, carbamazepine, oxcarbazepine and eslicarbazepine are not routinely used as first‐line agents and are not recommended by NICE as treatment options for generalised tonic–clonic seizures. Carbamazepine and oxcarbazepine may exacerbate seizures in people with absence or myoclonic seizures, including juvenile myoclonic epilepsy and are not recommended for these seizure types.^12^, ^46^ Guidelines from NICE recommend carbamazepine or oxcarbazepine as second‐line monotherapy options or as a first‐line add‐on option for focal seizures with or without evolution to bilateral tonic–clonic seizures. Eslicarbazepine is recommended as a second‐line add‐on option for focal seizures. At all stages of treatment (first‐line, second‐line and add‐on treatments), recommended alternatives to carbamazepine, oxcarbazepine and eslicarbazepine are available.^46^ Given the availability of alternative therapeutic options, pharmacogenetic testing should normally be undertaken prior to prescription unless the clinical benefits clearly outweigh the risks. In patients in whom alternative treatments are unsuccessful or contra‐indicated, pharmacogenetic testing should be requested as soon as possible if treatment with carbamazepine or related compounds is considered likely in the future. In paediatric practice there are some types of epilepsy where prescription of carbamazepine would be appropriate as first‐line and may be clinically urgent including focal seizures in infancy caused by gain‐of‐function mutations in sodium channel genes.^47^ In this case treatment can be initiated by specialists with testing undertaken at the same time, and reconsideration of treatment should a result be positive.

### 5.2 Bipolar disorder (also known as manic‐depressive psychosis or manic‐depressive illness)

A 2021 study of bipolar disorder prevalence and psychotropic medication utilisation in the United Kingdom and Hong Kong showed that the use of carbamazepine to treat bipolar disorder in the UK was low and declining in favour of alternative treatments.^48^ NICE guidelines no longer recommend carbamazepine as a treatment option for bipolar disorder in children, young people and adults.^49^ NICE recommends lithium as a first‐line long‐term pharmacological treatment for bipolar disorder and recommends antipsychotic therapies as second‐ and third‐line options. Fourth‐line treatment recommendations are to consider a combination of valproate with either an antipsychotic or lithium. Recent guidance from the MHRA restricting the use of valproate‐containing medicines for both epilepsy and bipolar disorder due to reproductive risks^15^ may result in an increase in the use of carbamazepine in individuals whose symptoms are not controlled on lithium or antipsychotic therapy, although the impact of this has yet to be seen at the time of publication. Given the availability of alternative therapeutic options, if carbamazepine treatment is being considered, pharmacogenetic testing for HLA‐B*15:02, HLA‐B*15:11 and HLA‐A*31:01 should be undertaken prior to prescription unless the clinical benefits clearly outweigh the risks.

### 5.3 Trigeminal neuralgia

Carbamazepine is recommended as first‐line treatment for trigeminal neuralgia in adults in guidelines from NICE^50^ and the Royal College of Surgeons of England.^51^ Carbamazepine may be initiated by specialists or in primary care and dentistry. If initiated by non‐specialists' expert advice or referral to a specialist pain or condition‐specific service is recommended if carbamazepine is not effective, not tolerated or is contra‐indicated. Pharmacogenetic testing for HLA‐B*15:02, HLA‐B*15:11 and HLA‐A*31:01 should be undertaken prior to prescription unless the clinical benefits clearly outweigh the risks. However, because of a lack of licensed alternatives that are recommended for prescribing in primary care, this may result in a delay in receipt of treatment to manage pain of up to 5–7 days whilst awaiting results. Based on a shared decision‐making model, a patient and prescriber may collaboratively decide whether to proceed with treatment immediately or to wait for a result, considering the specific risks based on the patient's ancestry.

### 5.4 Other indications

Oxcarbazepine is used off‐label for trigeminal neuralgia, and carbamazepine and oxcarbazepine are used off‐label for other types of neuropathic pain.^51^, ^52^ Further off‐label uses of carbamazepine may include medically assisted withdrawal from alcohol,^53^ restless legs syndrome,^54^ and management of agitation and aggression in dementia.^55^ Pharmacogenetic testing for HLA‐B*15:02, HLA‐B*15:11 and HLA‐A*31:01 should be undertaken prior to prescription unless there are no alternative treatment options available, and the clinical benefits clearly outweigh the risks.

### 5.5 Clinically urgent treatment before a test result is obtained

For all indications, if due to clinical urgency, treatment with carbamazepine, oxcarbazepine or eslicarbazepine is started before a test result is obtained, there is still benefit in requesting pharmacogenetic testing for HLA‐B*15:02, HLA‐B*15:11 and HLA‐A*31:01 at the time of treatment initiation since the onset of cutaneous adverse reactions is usually delayed. If a positive pharmacogenetic test result is received after treatment has started, then the prescribing clinician should review the prescription and discontinue treatment unless the clinical benefits clearly outweigh the risks.^1^

### 5.6 Communication between primary care and specialists

Where testing is ordered by a specialist, communication with primary care (and other clinical teams) is extremely important. This communication should provide the results of the *HLA* pharmacogenetic testing and whether carbamazepine, oxcarbazepine, eslicarbazepine and other medicines need to be avoided or discontinued based on the *HLA* pharmacogenetic test result. This decision, as well as recommendations for ongoing treatment, should be made by the specialist clinical teams who have ordered the pharmacogenetic test for the patient prior to initiation. Sharing this information to support ongoing patient care is important and relevant. SNOMED CT codes should be used to record the result in both hospital and primary care records (refer to Table 2). Where pharmacogenetic testing has been initiated in primary care, for example, for the treatment of trigeminal neuralgia, the result of the *HLA* test should be communicated to secondary care in any onward referrals.

**TABLE 2:** SNOMED‐CT codes for *HLA* genotypes associated with increased risk of severe cutaneous adverse reactions with carbamazepine and related compounds.

SNOMED CT code wording	SNOMED CT code ID
Human leukocyte antigen B*15:02 detected (finding)	738785009
Human leukocyte antigen B*15:02 not detected (finding)	738784008
Human leukocyte antigen A*31:01 detected (finding)	738783002
Human leukocyte antigen A*31:01 not detected (finding)	738782007
There are no SNOMED‐CT codes for HLA‐B*15:11 at present (new code requested 25/07/2025, expected in the April 2026 release)	

## 6 WHICH GENE(S), VARIANTS AND TURNAROUND TIME?

The following *HLA* alleles should be tested for when carbamazepine, oxcarbazepine or eslicarbazepine therapy is being considered:

1. HLA‐B*15:02
  [NG_023187.1:c.[5T>G; 11T>C; 44C>G; 45G>A; 103T>G; 106G>A; 142T>G; 204A>G; 205G>A; 206A>T; 209A>C; 213G>C; 222G>A; 272A>C; 277G>A; 280C>A; 282G>C; 283G>A; 292G>T; 353C>T; 355C>A; 363C>G; 369C>T; 409C>T; 419A>C; 463C>A; 477C>G; 539G>T; 559G>C; 560A>T; 603C>G; 605A>C; 610G>C; 618T>G; 636C>T; 693T>C; 756T>C; 900G>A; 916G>A; 985G>A; 1008T>C; 1046G>C]. Available at: https://www.ebi.ac.uk/cgi-bin/ipd/pl/hla/get_allele_hgvs.cgi?B*15:02:01:01
2. HLA‐B*15:11
  [NG_023187.1:c.[5T>G; 11T>C; 44C>G; 45G>A; 103T>G; 106G>A; 142T>G; 204A>G; 205G>A; 206A>T; 209A>C; 213G>C; 222G>A; 277G>A; 280C>A; 282G>C; 283G>A; 292G>T; 363C>G; 419A>C; 463C>A; 477C>G; 538C>T; 559G>C; 560A>T; 603C>G; 605A>C; 610G>C; 618T>G; 636C>T; 693T>C; 756T>C; 900G>A; 916G>A; 985G>A; 1008T>C; 1046G>C]. Available at: https://www.ebi.ac.uk/cgi-bin/ipd/pl/hla/get_allele_hgvs.cgi?B*15:11:01:01
3. HLA‐A*31:01
  [NM_002116.7:c.[41C>T; 97T>A; 98T>C; 238G>A; 243G>T; 282G>C; 290C>T; 363A>G; 413G>A; 448C>T; 502A>C; 524A>G; 527A>T; 555T>G; 633A>G; 642C>T; 649C>G; 651C>T; 652A>G; 691G>A; 808G>T; 829G>C; 870G>C; 899T>C; 945G>A; 952C>T; 964A>T; 967A>G; 987C>T; 992T>G; 1029T>C; 1033A>T; 1072G>A; 1077C>T]. Available at: https://www.ebi.ac.uk/cgi-bin/ipd/pl/hla/get_allele_hgvs.cgi?A*31:01:02:01

Patients are genotyped as positive and receive a positive pharmacogenetic test result if they have at least one copy of the above‐mentioned alleles. Pharmacogenetic testing for variant *HLA* alleles should be undertaken by laboratory testing, and the turnaround time within the laboratory should be 5 days or less. HLA typing should be performed to a minimum of two‐field resolution for HLA‐B*15:02, HLA‐B*15:11 and HLA‐A*31:01 alleles, and any ambiguities associated with the results should be reported. In addition, laboratories performing *HLA* genotyping for alleles associated with an increased risk of severe cutaneous adverse reactions to carbamazepine and related compounds should hold ISO 15189 accreditation and/or European Federation for Immunogenetics accreditation. In urgent situations, if the clinical benefit of starting treatment before testing is likely to outweigh the risk, then pharmacogenetic testing should be undertaken as soon as possible to guide the choice of ongoing treatment.

Genotyping test results should be displayed in reports as per current best practice guidance. This should then be recorded by the receiving clinician within the health record, ideally in the form of structured data, where this is available, for example, using SNOMED‐CT codes (Table 2). Therefore, laboratories may wish to highlight these codes in results reports.

## 7 CLINICAL ACTIONS BASED ON PHARMACOGENETIC TEST RESULTS

Prescribing recommendations should be guided by the pharmacogenetic test results as per Tables 3, 4, 5.

**TABLE 3:** Recommended clinical actions based on pharmacogenetic test results for epilepsy in children, young people and adults.

Carriers of *HLA‐B* or *HLA‐A* variant alleles associated with increased risk of severe cutaneous adverse drug reactions	Prescribing suggestions
HLA‐B*15:02‐positive and any HLA‐A*31:01 genotype	**Avoid carbamazepine, oxcarbazepine and eslicarbazepine.** ^a^ Consider alternative antiepileptic agents according to recommended treatment guidelines. Avoid the use of phenytoin and fosphenytoin if an alternative is possible (see notes below). Avoid the use of lamotrigine if an alternative is possible unless the benefit exceeds the risk (see notes below). Caution is advised with the use of phenobarbital and primidone (see notes below).
HLA‐A*31:01‐positive and HLA‐B*15:02‐negative	**Avoid carbamazepine, oxcarbazepine and eslicarbazepine** ^a^ **if an alternative is possible**. Only use carbamazepine, oxcarbazepine or eslicarbazepine after careful consideration of the benefits and risks. If it is not possible to use an alternative, increase monitoring and advise patients on what action to take if a skin rash occurs. Consider alternative antiepileptic agents according to recommended treatment guidelines.
HLA‐B*15:11‐positive	**Avoid carbamazepine, oxcarbazepine** ^b^ **and eslicarbazepine** ^a^ ^,^ ^b^ **if an alternative is possible**. Only use carbamazepine, oxcarbazepine or eslicarbazepine after careful consideration of the benefits and risk. If it is not possible to use an alternative, increase monitoring and advise patients on what action to take if a skin rash occurs. Consider alternative antiepileptic agents according to recommended treatment guidelines.

^a^ Eslicarbazepine acetate is a prodrug that is converted to the same active metabolite of oxcarbazepine, the active enantiomer of 10‐monohydroxy oxcarbazepine (S‐licarbazepine). Therefore, the same recommendations provided for oxcarbazepine have been applied to eslicarbazepine.

^b^ Although evidence of an association between HLA‐B*15:11 and hypersensitivity reactions is limited to carbamazepine only, recommendations to avoid have been extended to oxcarbazepine and eslicarbazepine based on the structural similarities between the compounds and the clinical cross‐reactivity.

**TABLE 4:** Recommended clinical actions based on pharmacogenetic test results in bipolar disorder in children, young people and adults.

Carriers of *HLA‐B* or *HLA‐A* variant alleles associated with increased risk of severe cutaneous adverse drug reactions	Prescribing suggestions
HLA‐B*15:02‐positive and any HLA‐A*31:01 genotype	**Avoid carbamazepine, oxcarbazepine and eslicarbazepine.** ^a^ Consider alternative agents according to recommended treatment guidelines. Avoid the use of lamotrigine if an alternative is possible unless the benefit exceeds the risk (see notes below).
HLA‐A*31:01‐positive and HLA‐B*15:02‐negative	**Avoid carbamazepine, oxcarbazepine and eslicarbazepine** ^a^ **if an alternative is possible**. Only use carbamazepine, oxcarbazepine or eslicarbazepine after careful consideration of the benefits and risk. If it is not possible to use an alternative, increase monitoring and advise patients on what action to take if a skin rash occurs. Consider alternative treatments according to recommended treatment guidelines.
HLA‐B*15:11‐positive	**Avoid carbamazepine, oxcarbazepine** ^b^ **and eslicarbazepine** ^a^ ^,^ ^b^ **if an alternative is possible**. Only use carbamazepine, oxcarbazepine or eslicarbazepine after careful consideration of the benefits and risk. If it is not possible to use an alternative, increase monitoring and advise patients on what action to take if a skin rash occurs. Consider alternative treatments according to recommended treatment guidelines.

^a^ Eslicarbazepine acetate is a prodrug that is converted to the same active metabolite of oxcarbazepine, the active enantiomer of 10‐monohydroxy oxcarbazepine (S‐licarbazepine). Therefore, the same recommendations provided for oxcarbazepine have been applied to eslicarbazepine. We have included both oxcarbazepine and eslicarbazepine in this table for the sake of completeness (given their structural similarity to carbamazepine) even though they are not used in bipolar disorder and are not licensed for this indication.

^b^ Although evidence of an association between HLA‐B*15:11 and hypersensitivity reactions is limited to carbamazepine only, recommendations to avoid have been extended to oxcarbazepine and eslicarbazepine based on the structural similarities between the compounds and the clinical cross‐reactivity.

**TABLE 5:** Recommended clinical actions based on pharmacogenetic test results in trigeminal neuralgia in children, young people and adults.

Carriers of *HLA‐B* or *HLA‐A* variant alleles associated with increased risk of severe cutaneous adverse drug reactions	Prescribing suggestions
HLA‐B*15:02‐positive and any HLA‐A*31:01 genotype.	**Avoid carbamazepine, oxcarbazepine and eslicarbazepine.** ^a^ Consider seeking expert advice from a specialist and consider early referral to a specialist pain service or a condition‐specific service. Consider alternative agents according to recommended treatment guidelines. Avoid the use of phenytoin and fosphenytoin if an alternative is possible (see notes below). Avoid the use of lamotrigine if an alternative is possible unless the benefit exceeds the risk (see notes below).
HLA‐A*31:01‐positive and HLA‐B*15:02‐negative	**Avoid carbamazepine, oxcarbazepine and eslicarbazepine** ^a^ **if an alternative is possible**. Only use carbamazepine, oxcarbazepine or eslicarbazepine after careful consideration of the benefits and risk. If it is not possible to use an alternative, increase monitoring and advise patients on what action to take if a skin rash occurs. Consider seeking expert advice from a specialist and consider early referral to a specialist pain service or a condition‐specific service. Consider alternative agents according to recommended treatment guidelines.
HLA‐B*15:11‐positive	**Avoid carbamazepine, oxcarbazepine** ^b^ **and eslicarbazepine** ^a^ ^,^ ^b^ **if an alternative is possible**. Only use carbamazepine, oxcarbazepine or eslicarbazepine after careful consideration of the benefits and risk. If it is not possible to use an alternative, increase monitoring and advise patients on what action to take if a skin rash occurs. Consider seeking expert advice from a specialist and consider early referral to a specialist pain service or a condition‐specific service. Consider alternative agents according to recommended treatment guidelines.

^a^ Eslicarbazepine acetate is a prodrug that is converted to the same active metabolite of oxcarbazepine, the active enantiomer of 10‐monohydroxy oxcarbazepine (S‐licarbazepine). Therefore, the same recommendations provided for oxcarbazepine have been applied to eslicarbazepine. We have included eslicarbazepine in this table for the sake of completeness (given its structural similarity to carbamazepine) even though it is not used in trigeminal neuralgia and is not licensed for this indication.

^b^ Although evidence of an association between HLA‐B*15:11 and hypersensitivity reactions is limited to carbamazepine only, recommendations to avoid have been extended to oxcarbazepine and eslicarbazepine based on the structural similarities between the compounds and the clinical cross‐reactivity.

### 7.1 Epilepsy in children, young people and adults

Where carbamazepine, oxcarbazepine or eslicarbazepine is the therapy of choice for people living with epilepsy, the prescribing recommendations reported in Table 3 should be considered.

The following points need to be considered when prescribing for patients with epilepsy:

- Clinicians should consult the relevant clinical guidelines from NICE^46^ when available as well as relevant local guidelines.
- Alternative treatment choice should be guided by the type of epilepsy, place in the treatment pathway, previous treatments tried, patient characteristics, comorbidities and contra‐indications.
- The following oral treatments may be considered as an alternative according to the NICE guideline ‘Epilepsies in children, young people and adults’^46^: levetiracetam, sodium valproate, zonisamide and lacosamide.
- The following add‐on medications may be considered according to the NICE guideline ‘Epilepsies in children, young people and adults’^46^: clobazam, levetiracetam, perampanel, sodium valproate, topiramate, brivaracetam, lacosamide, zonisamide, cenobamate, pregabalin, tiagabine and vigabatrin.
- The use of some antiseizure medications in children is off‐label.
- The use of some antiseizure medications in generalised tonic–clonic seizures is off‐label in children and adults.
- MHRA safety measures and precautionary advice should be followed for sodium valproate.^15^
- Do not use topiramate in women and girls of childbearing potential unless the conditions of the Pregnancy Prevention Programme are fulfilled.
- Local guidance and MHRA safety measures and advice regarding the risk of misuse and dependence should be followed for gabapentin and pregabalin.
- Caution is recommended when choosing an alternative agent. There is evidence of an association between HLA‐B*15:02 and other aromatic antiepileptic agents including lamotrigine, phenytoin and fosphenytoin. There is limited evidence of an association between HLA‐B*15:02 and phenobarbital. Primidone is partially metabolised in the liver into phenobarbital.^56^ Refer to the Section 11 for more details.
- Where a hypersensitivity reaction has occurred with one aromatic anticonvulsant agent, avoidance of the others is recommended unless the benefit exceeds risk.
- It is common to get a skin rash with carbamazepine, oxcarbazepine or eslicarbazepine. Most skin rashes are not serious. Patients should be advised to seek medical advice if a skin rash occurs and to go to A&E immediately if they experience a severe rash with flushing, blisters or ulcers as these can be signs of SJS.^57^
- The SmPCs for carbamazepine, oxcarbazepine and eslicarbazepine include information on contraindications and cautions, and it is important that these are followed. Prescribers should also be aware of drug–drug interactions with carbamazepine, oxcarbazepine and eslicarbazepine.

### 7.2 Bipolar disorder in children, young people and adults

Where carbamazepine is the therapy of choice in people living with bipolar disorder, the prescribing recommendations reported in Table 4 should be considered.

The following points need to be considered when prescribing for patients with bipolar disorder:

- Clinicians should consult the relevant clinical guidelines from specialist societies, Royal Colleges^58^ and NICE^49^ when available as well as relevant local guidelines.
- The following oral treatments may be considered as alternatives either alone or in combination according to the NICE guideline ‘Bipolar disorder: assessment and management’^49^: haloperidol, olanzapine, quetiapine, risperidone, fluoxetine, lithium, sodium valproate, asenapine and aripiprazole.
- The use of lithium in acute mania or hypomania is off‐label.
- The use of olanzapine or fluoxetine in bipolar depression is off‐label.
- The use of some antiseizure and psychotropic medications in children is off‐label.
- Semi‐sodium valproate has a UK marketing authorisation for long‐term pharmacological treatment to prevent relapse of acute mania in bipolar disorder in people who have had acute mania that has responded to treatment with semi‐sodium valproate.^49^
- The use of sodium valproate for long‐term pharmacological treatment to prevent relapse of bipolar disorder is off‐label, although its use is common in UK clinical practice.^49^
- MHRA safety measures and precautionary advice should be followed for valproate.^15^
- Caution is recommended when choosing an alternative agent. There is evidence of an association between HLA‐B*15:02 and other aromatic antiepileptic agents including lamotrigine. Refer to the Section 11 for more details.
- Where a hypersensitivity reaction has occurred with one aromatic anticonvulsant agent, avoidance of the others is recommended unless the benefit exceeds risk.
- It is common to get a skin rash with carbamazepine, oxcarbazepine or eslicarbazepine. Most skin rashes are not serious. Patients should be advised to seek medical advice if a skin rash occurs and to go to A&E immediately if they experience a severe rash with flushing, blisters or ulcers as these can be signs of SJS.^57^
- The SmPCs for carbamazepine, oxcarbazepine and eslicarbazepine include information on contraindications and cautions, and it is important that these are followed. Prescribers should also be aware of drug–drug interactions with carbamazepine, oxcarbazepine and eslicarbazepine.

### 7.3 Trigeminal neuralgia in children, young people and adults

Where carbamazepine or oxcarbazepine is the therapy of choice in people living with trigeminal neuralgia, the prescribing recommendations reported in Table 5 should be considered.

The following points need to be considered when prescribing for patients with trigeminal neuralgia:

- Clinicians should consult the relevant clinical guidelines from specialist societies, Royal Colleges^51^ and NICE^50^ when available as well as relevant local guidelines.
- The following oral treatments may be considered by specialists as an alternative according to the Royal College of Surgeons of England guidelines ‘Guidelines for the management of trigeminal neuralgia’^51^: baclofen, gabapentin and pregabalin.
- The following adjuvant medications may be considered according to the Royal College of Surgeons of England guidelines ‘Guidelines for the management of trigeminal neuralgia’^51^: topical lidocaine, sumatriptan subcutaneous injections, botulinum toxin type A and lidocaine subcutaneous infusion.
- Although not yet included in national clinical guidelines, lacosamide is a newer alternative option that may be considered for treatment refractory trigeminal neuralgia according to some local formularies and guidelines.^59^, ^60^, ^61^, ^62^, ^63^
- Carbamazepine is the only medicine licensed to treat trigeminal neuralgia in adults. It is important to note that the use of all other medicines to treat trigeminal neuralgia is off‐label.
- The use of some of the drugs mentioned in children is off‐label.
- Local guidance and MHRA safety measures and advice regarding the risk of misuse and dependence should be followed for gabapentin and pregabalin.^64^
- Caution is recommended when choosing an alternative agent. There is evidence of an association between HLA‐B*15:02 and other aromatic antiepileptic agents including lamotrigine, phenytoin and fosphenytoin. Refer to Section 11 for more details.
- Where a hypersensitivity reaction has occurred with one aromatic anticonvulsant agent, avoidance of the others is recommended unless the benefit exceeds risk.
- It is common to get a skin rash with carbamazepine, oxcarbazepine or eslicarbazepine. Most skin rashes are not serious. Patients should be advised to seek medical advice if a skin rash occurs and to go to A&E immediately if they experience a severe rash with flushing, blisters or ulcers as these can be signs of SJS.^57^
- The SmPCs for carbamazepine, oxcarbazepine and eslicarbazepine include information on contraindications and cautions, and it is important that these are followed. Prescribers should also be aware of drug–drug interactions with carbamazepine, oxcarbazepine and eslicarbazepine.

## 8 OTHER PHARMACOGENETICS GUIDELINES

In this section, prescribing recommendations provided by other well established international pharmacogenetics consortia are summarised for comparison with the UK CERSI‐PGx guidelines.

### 8.1 The Clinical Pharmacogenetics Implementation Consortium guideline

The Clinical Pharmacogenetics Implementation Consortium (CPIC) first issued a guideline in 2013 with a focus on the use of HLA‐B*15:02 genotyping to guide the prescription of carbamazepine.^4^ This was because there was high quality evidence linking HLA‐B*15:02 to an increased risk of carbamazepine‐induced SJS/TEN. CPIC recommended avoiding carbamazepine in HLA‐B*15:02‐positive subjects naïve to the drug. The strength of the recommendation was strong. Moreover, caution was (optionally) recommended for HLA‐B*15:02‐positive patients already on treatment with carbamazepine for more than 3 months without symptoms of SJS/TEN. In 2017, CPIC published an update: this included the use of HLA‐A*31:01 genotyping prior to the use of carbamazepine and recommendations to guide the prescription of oxcarbazepine.^1^ The main recommendations for HLA‐B*15:02‐positive subjects and carbamazepine remained the same, but they were extended to oxcarbazepine, that is, to avoid these two drugs if the subject was HLA‐B*15:02‐positive. Because cutaneous adverse reactions occur within the first 3 months of therapy, if the patient was previously treated with either carbamazepine or oxcarbazepine consistently for longer than 3 months without developing cutaneous adverse reactions, then the updated recommendation was that clinicians can cautiously consider the reuse of these drugs in the future.^1^ If the patient is HLA‐A*31:01‐positive (but HLA‐B*15:02‐negative), CPIC recommends avoiding carbamazepine in drug naïve patients. The strength of this recommendation is strong. However, if there are no other treatment options available, clinicians can consider the use of carbamazepine with increased monitoring. Finally, the use of carbamazepine with caution can be considered in HLA‐A*31:01‐positive patients who have previously been treated with carbamazepine consistently for longer than 3 months without developing hypersensitivity. The strength of these last two recommendations is optional.^1^ Despite the lack of evidence with the other aromatic antiseizure medications, CPIC cautioned against the use of these drugs because of the known risk of cross‐reactivity.

### 8.2 The Dutch Pharmacogenetics Working Group guideline

The Dutch Pharmacogenetics Working Group (DPWG) guideline on the use of *HLA* testing before carbamazepine therapy states that there is strong evidence implicating HLA‐B*15:02 in predisposing to carbamazepine‐induced SJS/TEN.^5^ Six meta‐analyses have been published, reporting an increased risk of carbamazepine induced SJS/TEN in HLA‐B*15:02‐positive subjects (OR = 27–138).^21^, ^40^, ^65^, ^66^, ^67^, ^68^ The DPWG recommends avoidance of carbamazepine in HLA‐B*15:02‐positive subjects. In addition, the guideline also highlights that an association exists between HLA‐B*15:02 and SJS/TEN induced by other anticonvulsant drugs, including oxcarbazepine, phenytoin and lamotrigine, although the risk for SJS/TEN in HLA‐B*15:02‐positive carriers is approximately 5‐ to 10‐fold lower for these drugs than with carbamazepine (see Section 11 for more details).

The DPWG also recognises the increased risk of severe cutaneous adverse reactions in HLA‐B*15:11‐positive individuals. Prescribing recommendations for these subjects are to carefully weigh the risk of SJS/TEN against the benefits and avoid carbamazepine if an alternative agent is available. If there is no alternative to carbamazepine, clinicians should advise patients to report any skin rash immediately. Similarly, for HLA‐A*31:01 carriers, the DPWG advises to carefully consider the risk of DRESS and SJS/TEN against the benefits and avoid carbamazepine if the use of an alternative drug is possible. If it is not possible and carbamazepine has to be used, patients should be advised to report any skin rash immediately.^5^ Similarly for the other anticonvulsant drugs, including oxcarbazepine the DPWG recommendation is to choose an alternative agent. If an alternative is not available, it is recommended to advise the patient to report any rash immediately.

### 8.3 The Canadian Pharmacogenomics Network for Drug Safety guideline

In 2014, the Canadian Pharmacogenomics Network for Drug Safety (CPNDS) clinical recommendation group published a guideline for the use of both HLA‐B*15:02 or HLA‐A*31:01 alleles to optimise the prescription of carbamazepine.^69^ Their recommendation was to avoid carbamazepine in drug‐naïve patients who were carriers of at least one HLA‐B*15:02 or HLA‐A*31:01 allele.

### 8.4 The French National Network of Pharmacogenetics guideline

The 2025 guideline of the Société Francophone d'Histocompatibilité et d'Immunogénétique (SFHI) on HLA genotyping includes recommendations on HLA‐B*15:02 and HLA‐A*31:01 genotyping in relation to carbamazepine.^70^ This guideline is included in the ClinPGx database as guidance provided by the French National Network of Pharmacogenetics (RNPGx).^71^ The SFHI recommends genotyping for HLA‐B*15:02 and HLA‐A*31:01 alleles upon request. The HLA‐B*15:02 test is particularly recommended for patients of Asian descent, like Southeast Asian or Chinese ancestry, before starting treatment whereas HLA‐A*31:01 genotyping is not routinely recommended before initiating treatment. The level of these recommendations is strong. Carbamazepine is contraindicated in HLA‐B*15:02‐positive patients unless there are no other alternatives available, and strict clinical monitoring is required. In HLA‐A*31:01‐positive patients, a re‐evaluation of the risk–benefit ratio is recommended.

Table 6 provides a summary of current PGx guidelines.

**TABLE 6:** Summary of therapeutic recommendations based on different guidelines.

Pharmacogenetic test result (HLA genotype)	CPIC	DPWG	CPNDS	RNPGx	UK CERSI‐PGx
	Therapeutic recommendations (classification of the recommendations)	Therapeutic recommendations^c^	Therapeutic recommendations^e^ (classification of the recommendations)	Therapeutic recommendations^g^	Therapeutic recommendations
HLA‐B*15:02‐positive	If the patient is treatment naïve,^a^ do not use carbamazepine or oxcarbazepine^b^ (strong).	Avoid carbamazepine.^d^ Avoid oxcarbazepine if an alternative is possible.^d^	An alternative to carbamazepine should be used as first‐line therapy^f^ (strong). Avoid oxcarbazepine as first‐line therapy^f^ (optional).	Carbamazepine is contraindicated unless no alternative is available, strict clinical monitoring required.	Avoid carbamazepine, oxcarbazepine and eslicarbazepine.
HLA‐A*31:01‐positive	If the patient is treatment naïve^a^ and alternative agents are available, do not use carbamazepine (strong).	Avoid carbamazepine if an alternative is possible.	An alternative to carbamazepine should be used as first‐line therapy^f^ (strong). Avoid oxcarbazepine as first‐line therapy^f^ (optional).	Increased risk with carbamazepine. Re‐evaluate risk–benefit ratio.	Avoid carbamazepine, oxcarbazepine and eslicarbazepine if an alternative is possible.
HLA‐B*15:11‐positive	No recommendation	Avoid carbamazepine if an alternative is possible.	No recommendation	No recommendation	Avoid carbamazepine, oxcarbazepine and eslicarbazepine if an alternative is possible.

^**a**^ Patients who have not previously taken carbamazepine for >3 months without hypersensitivity reactions.^1^

^b^ The Clinical Pharmacogenetics Implementation Consortium (CPIC) recommends that in HLA‐B*15:02‐positive patients, phenytoin and fosphenytoin should not be used and advises caution with other aromatic anticonvulsants including eslicarbazepine, lamotrigine and phenobarbital.^1^

^c^ The Dutch Pharmacogenetics Working Group (DPWG) considers: HLA‐B*15:02 genotyping of patients of Asian descent, other than Japanese before starting carbamazepine or oxcarbazepine to be beneficial for drug safety; HLA‐A*31:01 genotyping and HLA‐B*15:11 genotyping of patients of Han Chinese, Korean, Thai or Japanese descent before starting carbamazepine to be beneficial for drug safety. DPWG recommends considering genotyping these patients before (or directly after) drug therapy has been initiated to guide drug selection.^5^

^d^ The DPWG recommends that in HLA‐B*15:02‐positive patients, lamotrigine and phenytoin should also be avoided if an alternative is possible.^5^

^e^ The Canadian Pharmacogenomics Network for Drug Safety (CPNDS) recommends pharmacogenetic testing for HLA‐B*15:02 for all carbamazepine‐naïve patients before initiation of carbamazepine therapy. The level of recommendation is strong in patients originating from populations where HLA‐B*15:02 is common, optional in patients originating from populations where HLA‐B*15:02 is rare. The CPNDS advises that the safest option would be to offer HLA‐B*15:02 genotyping to all patients, irrespective of their ancestry. HLA‐B*15:02 genotyping is also recommended in patients with a previous hypersensitivity reaction potentially related to carbamazepine as part of the differential diagnosis and to guide future therapy and in patients for whom no alternative treatment options are available. Pharmacogenetic testing for HLA‐A*31:01 is recommended for all carbamazepine‐naive patients before initiation of carbamazepine therapy. The level of recommendation is moderate in all patients.^69^

^f^ The CPNDS recommends first choice of alternative medications should be given to those that are structurally different from carbamazepine. If structurally different medications are not effective or tolerated, aromatic antiseizure medicines other than carbamazepine or oxcarbazepine should be used.^69^

^g^ The Société Francophone d'Histocompatibilité et d'Immunogénétique (SFHI) recommends genotyping for HLA‐B*15:02 and HLA‐A*31:01 alleles upon request prior starting carbamazepine treatment.^70^ This guideline is included in the ClinPGx database as a guidance provided by the French National Network of Pharmacogenetics (RNPGx).^71^ HLA‐B*15:02 genotyping is particularly recommended for patients of Asian descent (Southeast Asian or Chinese ancestry) before starting a treatment whereas HLA‐A*31:01 genotyping is not routinely recommended before initiating treatment. The level of these recommendations is strong (Grade 1A).

### 8.5 Other guidelines

#### 8.5.1 The Royal College of Psychiatrists college report on the role of genetic testing in mental health settings

This report recommended screening for variant *HLA‐A* and *HLA‐B* alleles before prescribing certain mood stabilisers (e.g., carbamazepine) in ‘particular’ ethnic groups (e.g., East Asians).^58^

#### 8.5.2 The Royal College of Surgeons of England guidelines for the management of trigeminal neuralgia

This guideline recommended that HLA‐B*15:02 allele testing should be undertaken in individuals of Han Chinese or Thai origin who are being considered for treatment with carbamazepine or oxcarbazepine for trigeminal neuralgia.^51^

## 9 HEALTH ECONOMIC EVALUATION

The economic evidence relates to HLA‐B*15:02 and HLA‐A*31:01 pharmacogenetic testing prior to treatment with carbamazepine. No cost utility analyses were identified for HLA‐B*15:11 testing for any indication, and none of the evaluations considered oxcarbazepine or eslicarbazepine.

### 9.1 Epilepsy

Evidence for the cost‐utility of testing for HLA‐B*15:02 prior to initiating carbamazepine in people with epilepsy comes from 10 economic evaluations published between 2012 and 2024, covering a range of countries, healthcare systems and populations. These compared single gene tests to standard care, with carriers of HLA‐B*15:02 prescribed a range of alternative antiseizure medications across the studies: phenytoin, lamotrigine, valproate or levetiracetam. Most evaluations used decision‐analytic models, combining decision tree frameworks with Markov simulations that considered seizure control as well as SJS/TEN and adopted perspectives ranging from healthcare provider, national health system to societal viewpoints.

The findings suggest that single gene tests are cost‐effective in the United Kingdom,^72^ Thailand,^73^ Indonesia^74^ and Malaysia^75^; in Chinese and Malay populations in Singapore^76^; and amongst patients of Asian ancestry in the United States^77^ and Australia.^78^ However, HLA‐B*15:02 testing does not appear to be cost‐effective in Hong Kong,^79^ amongst Indian Singaporeans^76^ and according to earlier Malaysian^80^ and Indonesian studies.^81^

Differences between study results are attributable to model structures and assumptions such as in relation to time horizon of analysis and choice of comparator/alternative treatment and to variations in input parameters—mainly, HLA‐B*15:02 allelic frequency, the cost of testing and the cost of alternative treatments. For instance, Chong et al.'s update^75^ of their 2017 analysis^80^ included revised (higher) prevalence of HLA‐B*15:02 and costs (lower) of testing resulting in testing becoming cost‐effective. Dong et al.^76^ reported that to avoid one case of SJS/TEN, 142 Chinese, 28 Malay or 833 Indian Singaporean patients would need to be genotyped, reflecting differences that result from HLA‐B*15:02 allelic frequencies and incidence of SJS/TEN and which are linked directly to cost‐effectiveness.

The cost‐effectiveness of single gene HLA‐A*31:01 testing (at £142 per test) was assessed in a UK analysis^42^ in which a Markov model was used to estimate total costs and quality‐adjusted life‐years (QALYs) over a lifetime to account for differences in the effectiveness of antiseizure medications and the long‐term consequences of cutaneous adverse drug reactions. The model represented a northern European population, assumed carriers of HLA‐A*31:01 are prescribed lamotrigine, patients who experience adverse drug reactions are prescribed valproate. The results indicated that 125 patients need to be tested to prevent one case of cutaneous adverse drug reaction (comprising mainly of MPE) and 3667 to prevent one case of HSS/SJS/TEN. The incremental cost‐effectiveness ratio was £12,808 per QALY gained, indicating that testing for HLA‐A*31:01 is cost‐effective for the National Health Services in the United Kingdom.

Testing for HLA‐A*31:01 remained cost‐effective at £15,638 per QALY gained when included within a £50, 6‐HLA gene panel.^72^ However, when reported as an incidental finding, HLA‐B*15:02 was not cost‐effective based on model inputs from Chen et al.^79^, which reported low adherence to prescribing guidance. Current service provision across the United Kingdom^82^ costs £150–£170 for comprehensive *HLA* class I (A, B and C) and class II (DR, DQ and DP) DNA typing by next‐generation sequencing, regardless of how many variants are initially requested or reported (Dr Deborah Pritchard Welsh Blood Service, personal communication, 15 September 2025). An update and revision of Plumpton et al.,^72^ focusing only on carbamazepine and HLA‐A*31:01 and HLA‐B*15:02, based on data from Gu et al.^78^ and with a test price of £160, resulted in an incremental cost‐effectiveness ratio of £17,053 per QALY gained (2024 prices)—indicating that testing for both alleles would represent good value for money.

An analysis from The Netherlands^83^ that adopted a short time horizon of 1 year found that preprescription testing for HLA‐B*15:11, HLA‐B*15:02 or HLA‐A*31:01 prior to carbamazepine or HLA‐B*15:02 prior to oxcarbazepine was not cost‐effective. However, this was based on an outcome of prevented deaths, which differs appreciably from more conventional measures of life years or quality‐adjusted life years gained and are therefore incomparable.

### 9.2 Bipolar disorder

No economic evaluations were identified for *HLA‐A* or *HLA‐B* pharmacogenetic testing in relation to the use of carbamazepine and related drugs for the management of bipolar disorder.

### 9.3 Trigeminal neuralgia

Rattanavipapong et al.^73^ reported that the provision of HLA‐B*15:02 screening prior to starting treatment with carbamazepine was cost‐effective in the context of managing neuropathic pain in Thailand. Their analysis assumed gabapentin as the alternative treatment, an allelic prevalence of 15.5%, and was based on a model that considered the lifetime costs and sequelae of SJS/TEN but not of pain management.

## 10 REGULATORY CONSIDERATIONS

### 10.1 Summary of Product Characteristics (SmPC)

The UK SmPCs for carbamazepine, oxcarbazepine and eslicarbazepine^12^, ^13^, ^14^ all highlight the association between HLA‐B*15:02 or HLA‐A*31:01 and severe cutaneous adverse drug reactions in Section 4.4 (*Special warnings and precautions in use*). All three SmPCs recommend that in individuals of Han Chinese or Thai origin who test positive for HLA‐B*15:02 treatment should not be started unless there is no other therapeutic option, and in individuals of European or Japanese origin who test positive for HLA‐A*31:01, treatment should only be considered if the benefits are thought to outweigh the risks. The SmPC for carbamazepine also states in Section 4.2 *(Posology and method of administration)* that before deciding to initiate treatment, patients of Han Chinese and Thai origin should wherever possible be screened for HLA‐B*15:02. All three SmPCs recommend in Section 4.4 (*Special warnings and precautions in use)* that testing ‘genetically at‐risk populations’ for HLA‐B*15:02 should be considered but that there is insufficient data supporting a recommendation for HLA‐A*31:01 screening before starting treatment with carbamazepine or chemically related compounds.

### 10.2 MHRA drug safety updates

#### 10.2.1 MHRA drug safety update 2008

In April 2008, the MHRA published a Drug Safety Update^84^ advising of the association between carbamazepine‐induced SJS and HLA‐B*15:02 and recommended that individuals of Han Chinese, Hong Kong Chinese or Thai origin should be screened for HLA‐B*15:02 before prescribing carbamazepine and that those who test positive should not start treatment unless the benefits clearly outweigh the risks.

#### 10.2.2 MHRA drug safety update 2014

A further 2014 MHRA Drug Safety Update^85^ advised that the presence of the HLA‐A*31:01 allele may increase the risk for carbamazepine‐induced skin reactions in patients of European descent or Japanese origin and if patients are known to be positive for HLA‐A*31:01 they should only receive carbamazepine, oxcarbazepine or eslicarbazepine after careful consideration of the benefits and risk.

## 11 OTHER CONSIDERATIONS

This section provides an overview of the evidence of an association between *HLA* biomarkers and toxicity with other aromatic antiepileptic agents that may be considered as alternative therapeutic options to carbamazepine, oxcarbazepine or eslicarbazepine. Clinicians should also be aware that there may be other coprescribed medications for other indications that this section will not cover and for which pharmacogenetics‐based guidance may be available through other sources.

### 11.1 Association between HLA‐B*15:02 and other aromatic antiepileptic agents that may be considered as an alternative to carbamazepine, oxcarbazepine and eslicarbazepine

There is evidence of an association between HLA‐B*15:02 and other aromatic antiepileptic agents including phenytoin, fosphenytoin and lamotrigine. Fosphenytoin is a water‐soluble prodrug that is converted to phenytoin to exert its clinical actions, and thus, evidence regarding phenytoin is relevant to fosphenytoin as well. There is limited evidence of an association between HLA‐B*15:02 and phenobarbital. Primidone is an anticonvulsant largely metabolised into two main metabolites phenobarbital and phenylethylmalonamide (PEMA),^56^ and thus, evidence regarding phenobarbital is relevant to primidone as well. However, because of the aromatic structures of these drugs, clinical cross‐reactivity for skin rashes has been reported in 40–80% of patients.^86^ Although evidence was mainly collected from Asian populations due to the higher frequency of the allele in this ethnic group, HLA‐B*15:02 may also occur in other populations or in patients that fail to report Asian ancestry in their families.^86^, ^87^

#### 11.1.1 Phenytoin and fosphenytoin

The literature review covering the period between 2005 and 2022 carried out by the DPWG^5^ on the association between HLA‐B*15:02 and phenytoin‐induced SJS/TEN included meta‐analyses, case–control studies that had at least 10 cases with severe cutaneous adverse events and studies and case reports investigating possible alternatives in the risk analysis. The incidence of SJS/TEN in subjects treated with phenytoin is estimated to be 0.069% in European and 0.24% in Asian treatment naïve patients.^5^, ^88^ The reactions tend to develop within the first 2–3 months of therapy. Four meta‐analyses of case–control studies, all performed in Asians, have consistently shown a significant association between HLA‐B*15:02 and phenytoin‐induced SJS/TEN. A meta‐analysis of 10 studies found an OR of 3.63 (95% CI, 2.15–6.13; *p* < 0.001) for the development of SJS/TEN in HLA‐B*15:02‐positive patients.^89^ Similarly, a previous meta‐analysis of seven studies found an OR of 3.60 (95% CI, 1.59–8.15; *p* = 0.001) for SJS/TEN in the presence of the HLA‐B*15:02 allele.^65^ Bloch and colleagues^67^ highlighted that one out of four studies included in their analysis did not show an increased risk of severe cutaneous adverse reactions in HLA‐B*15:02‐positive patients treated with phenytoin (OR 0.35; 95% CI, 0.03–3.9; *p* = 0.35).^90^ Likewise, one meta‐analysis of two studies including a cohort of Thai and a cohort of Japanese patients did not confirm the relevance of the association between HLA‐B*15:02 and SJS/TEN in response to phenytoin.^91^ In addition, the DPWG evaluated the outcomes of 10 case–control studies with at least 10 cases of phenytoin‐induced severe cutaneous adverse events. Amongst these studies, four found an increased risk for HLA‐B*15:02 carriers to develop SJS/TEN with ORs ranging between 3.5 and 6.5.^91^, ^92^, ^93^, ^94^ However, 6/10 case–control studies failed to replicate this association.^65^, ^90^, ^95^, ^96^, ^97^, ^98^ Taken together, the evidence suggests that there is a gene–drug interaction between HLA‐B*15:02 and severe cutaneous reactions to phenytoin, but the risk is less than with carbamazepine or its analogues.

We searched for additional studies published between 2022 and 2025, using the following search strings (phenytoin) AND (*HLA*‐B) AND (SJS) AND (toxic epidermal necrolysis) in PubMed and found two additional meta‐analyses. Both studies confirmed the increased risk of phenytoin‐induced SJS/TEN in HLA‐B*15:02‐positive subjects. The first, a meta‐analysis of eight studies including 128 cases and 438 controls, found an OR of 2.45 (95% CI, 1.52–3.95; *p* < 0.0002; *I* ^2^ = 43%),^99^ whereas the second meta‐analysis of 13 studies which included 296 cases and 1264 controls showed an OR of 3.00 (95% CI, 1.65–5.47; *p* < 0.01; *I* ^2^ = 64%).^100^ It is also worth noting that, in 2021, CPIC published an updated guideline on the use of *CYP2C9* and HLA‐B*15:02 pharmacogenetic biomarkers to optimise the clinical use of phenytoin. CPIC recommends avoiding the use of phenytoin and fosphenytoin in phenytoin‐naïve patients positive for the HLA‐B*15:02 allele, which concurs with our recommendation. In addition, they recommended avoiding the use of carbamazepine and oxcarbazepine as alternative drugs in these patients. The strength of this recommendation is ‘strong’.^87^

It is important to note that an association has also been described between the loss‐of‐function variant CYP2C9*3 and severe cutaneous adverse drug reactions with phenytoin (OR 12; 95% CI, 6.6–20; *p* = 1.1 × 10^−17^).^101^ The association with *CYP2C9* allelic variants has not been described with carbamazepine or its analogues. This is consistent with the fact that phenytoin, but not carbamazepine, is metabolised by CYP2C9.

#### 11.1.2 Lamotrigine

With respect to the association between HLA‐B*15:02 and lamotrigine‐induced SJS/TEN, the literature review carried out by the DPWG included studies published between 2010 and 2021.^5^ Only case–control studies with more than five cases with severe cutaneous adverse events in the risk analysis were considered. The incidence of SJS/TEN was reported to be 0.1% in adult patients, and the incidence of cutaneous eruptions requiring hospital admission was 0.3%–1% in paediatric subjects.^5^ Lamotrigine‐induced hypersensitivity reactions generally develop between 2 weeks and 3 months after the initiation of therapy. Data from four meta‐analyses,^67^, ^93^, ^94^, ^102^ one case–control study including 28 SJS/TEN cases in Iranians,^103^ and two pooled case–control studies including seven SJS/TEN cases in a Han Chinese population^104^ have shown an increased risk of developing SJS/TEN in HLA‐B*15:02‐positive patients exposed to lamotrigine. The OR ranged between 2.4 and 7.9 in these different studies, with the meta‐analyses showing reduced OR values with the increased number of cases included in the analysis.^5^ There were five studies showing no significant association between HLA‐B*15:02 and lamotrigine‐induced SJS/TEN. These studies include a meta‐analysis with seven Han Chinese SJS/TEN cases^94^ and four case–control studies including between six and 22 Chinese SJS/TEN cases.^65^, ^97^, ^105^, ^106^ We searched for additional studies published between 2021 and 2025, using the following search strings (lamotrigine) AND (HLA‐B) AND (SJS) AND (toxic epidermal necrolysis) in PubMed and found three additional meta‐analyses. The review by Tham and collaborators^100^ reports only the results of three previous meta‐analyses on the topic, highlighting that the ORs for development of SJS/TEN in HLA‐B*15:02‐positive subjects treated with lamotrigine ranged from 2.55 (95% CI, 1.29–5.04) to 4.83 (95% CI, 1.27–18.45). The other two meta‐analyses confirmed the increased risk of lamotrigine‐induced SJS/TEN in HLA‐B*15:02‐positive subjects. The first meta‐analysis of four studies included 46 cases and 118 controls and found an OR of 3.83 (95% CI, 1.49–9.86; *p* < 0.005; *I* ^2^ = 0%).^99^ The second meta‐analysis of 10 studies included 85 cases and 378 controls and found an OR of 2.88 (95% CI, 1.60–5.17; *p* < 0.0004; *I* ^2^ = 0%).^107^ The trend to reduced OR by increasing the number of cases studied seems to be confirmed.

In conclusion, there is some evidence of an association between lamotrigine‐induced SJS/TEN and HLA‐B*15:02, but this is not strong and may be confounded because of sequential use of lamotrigine in individuals who had not recovered from an episode of SJS/TEN after the use of carbamazepine. Thus, through an abundance of caution, we recommend the avoidance of lamotrigine unless the benefit exceeds risk, and an alternative agent is not available.

#### 11.1.3 Phenobarbital and primidone

In relation to phenobarbital induced SJS/TEN and HLA‐B*15:02, data are limited. Cheung and collaborators^93^ included two cases of SJS/TEN due to phenobarbital amongst their 55 cases but did not find any association with the HLA‐B*15:02 allele. A recent meta‐analysis including two studies with a total of 29 cases of phenobarbital‐induced SJS/TEN and 59 tolerant patients did not show an increased risk in the presence of the HLA‐B*15:02 allele (OR 0.93; 95% CI, 0.24–3.54; *p* = 0.91; *I* ^2^ = 54%).^99^ In a recent report, only one patient out of three cases of SJS/TEN induced by phenobarbital was HLA‐B*15:02‐positive.^108^ Other studies have included a limited number of both adult and paediatric cases of SJS/TEN due to phenobarbital and failed to find a significant gene–drug association, although they were probably underpowered.^65^, ^90^, ^109^, ^110^, ^111^, ^112^ However, because of the clinical cross‐reactivity between carbamazepine and phenobarbital, we recommend caution with the use of phenobarbital, and by extension, primidone, in a patient who is HLA‐B*15:02‐positive, unless the benefit exceeds risk.

### 11.2 Association between HLA‐A*31:01 and other aromatic antiepileptic agents that may be considered as an alternative to carbamazepine, oxcarbazepine and eslicarbazepine

In relation to HLA‐A*31:01 and risk of hypersensitivity reactions, the association has only been convincingly reported with carbamazepine and not with the other aromatic anticonvulsants, including lamotrigine, phenytoin, fosphenytoin and phenobarbital. However, because of the evidence supporting clinical cross reactivity amongst these drugs, caution is advised unless alternative agents are not available, and the benefit is considered to exceed the risk.

### 11.3 Association between HLA‐B*15:02 and lacosamide that may be considered as an alternative to carbamazepine

Lacosamide is the leading compound of a novel class of antiepileptic drugs called functionalised amino acids. It is licensed for use as monotherapy in the treatment of partial onset seizures with or without secondary generalisation in adults, adolescents and children from 2 years of age. However, as mentioned in Section 7.3, lacosamide is also used off‐label in the treatment of refractory trigeminal neuralgia.^59^, ^60^, ^61^, ^62^, ^63^ A pilot dose escalation study found that 200‐ or 400‐mg lacosamide twice daily was safe and effective in patients with trigeminal neuralgia.^59^ Sleepiness, dizziness and mood instability were reported as adverse events. Three patients who were positive for the HLA‐B*15:02 allele did not experience any cutaneous reactions. Overall, this is consistent with the known safety profile of lacosamide in epilepsy, with central nervous system adverse events being most reported in a pooled analysis of three pivotal phase II/III randomised trials^113^. Cutaneous eruptions occurred at a similar rate in the lacosamide group (27/944, 2.9%) in comparison to placebo (11/364, 3.0%). None of the patients developed severe cutaneous adverse drug reactions.^113^

It is, however, important to note that cutaneous adverse reactions have been reported in patients treated with lacosamide. A 36‐year‐old woman with unknown HLA status developed a cutaneous eruption 2 days after lacosamide escalation from 50 to 100 mg twice daily. She had previously experienced a similar reaction with lamotrigine.^114^ Another patient with unknown HLA status and a history of a ‘skin rash’ with phenytoin developed DRESS after lacosamide initiation. Immediately preceding lacosamide‐induced DRESS, he had developed a maculopapular eruption together with fever 16 days after the start of phenobarbital. The authors postulated cross‐reactivity between phenytoin, phenobarbital and lacosamide because of their common aromatic ring structure.^115^ Cases of SJS/TEN have also been reported in patients treated with lacosamide^116^, ^117^ for epilepsy, one of whom had also previously developed a cutaneous eruption with lamotrigine.^116^ Four cases of cutaneous eruptions induced by lacosamide were also recently reported in Chinese children with epilepsy, two of which occurred in HLA‐B*15:02‐positive patients.^118^ A literature review of 13 cases of lacosamide induced cutaneous eruptions (including the four cases described above) found that eight of the affected patients were previously treated with various antiseizure medications, seven of whom had tried four or more these drugs. The HLA‐B*15:02 status was known in four of the cases. Cutaneous eruptions developed within 1–10 days after lacosamide initiation with duration of 2–37 days. Two patients had a history of hypersensitivity to other aromatic antiseizure medications.^118^ Taken together, these data suggest a potential risk of cutaneous hypersensitivity reactions to lacosamide, which like carbamazepine, can vary in severity. However, there is insufficient evidence of an association with HLA‐B*15:02 although there does seem to be a risk of cross‐reactivity with other aromatic antiseizure medications, but the mechanisms are not clear.

## 12 RESEARCH RECOMMENDATIONS

During the development of these guidelines, it became evident that several areas lack sufficient supporting evidence. In the following section, we outline research recommendations intended to highlight key opportunities for further investigation. Although not exhaustive, these suggestions offer insight into where additional research could strengthen the evidence base for the use of *HLA* genotyping with carbamazepine, oxcarbazepine and eslicarbazepine.

### 12.1 HLA variation and impact on adverse drug reactions

- Further studies are needed to assess the association between eslicarbazepine‐ or oxcarbazepine‐induced hypersensitivity reactions and clinically relevant *HLA* alleles.
- Evidence in neuropathic pain or trigeminal neuralgia populations is limited, and thus, studies generating data in trigeminal neuralgia are necessary considering that carbamazepine is a first‐line treatment for this condition.
- Further studies are needed to assess the association between severe cutaneous adverse drug reactions and clinically relevant *HLA* alleles with other aromatic antiepileptic agents including phenytoin, fosphenytoin, lamotrigine and phenobarbital.
- Given the rarity of these serious reactions, as well as the declining use of carbamazepine and its analogues, clinical studies may not be possible. Therefore, more extensive laboratory based and molecular modelling studies that evaluate the mechanism(s) of cross‐reactivity would provide alternative and valuable evidence on which to provide recommendations.

### 12.2 Clinical utility of testing

- Further studies are needed to assess the clinical utility of HLA‐B*15:11 testing to identify patients at increased risk of severe cutaneous adverse reactions with carbamazepine and related compounds.

### 12.3 Health economic evidence

- Further economic evidence is required to evaluate the cost effectiveness of HLA‐B*15:02 and HLA‐A*31:01 testing for carbamazepine in indications other than epilepsy for oxcarbazepine and eslicarbazepine in any indications and HLA‐B*15:11 testing for any drug or indication.

## AUTHOR CONTRIBUTIONS

Munir Pirmohamed chaired the writing committee for the development of the guideline. All authors were members of the writing committee. *Conceptualisation:* Lucy Galloway, Cinzia Dello Russo and Munir Pirmohamed. *Methodology:* Lucy Galloway, Cinzia Dello Russo and Munir Pirmohamed. *Investigation:* Lucy Galloway, Cinzia Dello Russo and Munir Pirmohamed contributed to the background and evidence overview. Lucy Galloway, Nicholas Bass, Elvira Bramon, Natalie Curley, Sarah Curran, Helen Cross, Helen Davies, Jana De Villiers, William Evans, Bernhard Frank, Alice Groves, Judith Hayward, Jon Higham, Anthony G Marson, Ailsa McLellan, Seth Mensah, Francis O'Neill, Jane Sarginson, Sanjay M Sisodiya, Jill Swan, Joanna M Zakrzewska and Munir Pirmohamed contributed to the development of recommended indication for pharmacogenetic testing and integration of pharmacogenetic testing into existing clinical pathways and clinical action based on genotype. Lucy Galloway worked on the summary tables for the clinical recommendations. Lucy Galloway and Cinzia Dello Russo inputted on the details of pharmacogenetic testing. Dyfrig A Hughes and Shwe Sin Kyaw curated the Health Economics section. Lucy Galloway and Cinzia Dello Russo reviewed the evidence related to other pharmacogenetic guidelines. Cinzia Dello Russo contributed to the review of the associations between HLA alleles and toxicity of other aromatic antiepileptic agents and lacosamide. All authors contributed to the research recommendations section. *Resources and funding acquisition:* Munir Pirmohamed. *Writing*—*original draft*: Lucy Galloway, Cinzia Dello Russo, Dyfrig A Hughes and Munir Pirmohamed. *Writing*—*review and editing*: All authors. Lucy Galloway, Cinzia Dello Russo and Munir Pirmohamed revised the guideline according to the feedback received after the consultation process. Dyfrig A Hughes addressed comments related to the health economics section. All authors approved the revised version of the guideline and comments provided in the Supporting Information.

## CONFLICT OF INTEREST STATEMENT

The conflict of interest statements are documented in Table S1.

## Supporting information


**Table S1.** Competence, affiliations and disclosure of conflicts of interest of the writing committee of the CERSI‐PGx Guideline for HLA genotype testing for carbamazepine, oxcarbazepine and eslicarbazepine.
**Table S2.** Comments received during the consultation period by various organ on the CERSI‐PGx Guideline for *HLA* genotype testing for carbamazepine, oxcarbazepine and eslicarbazepine and responses by the UK CERSI PGx writing committee.

## Data Availability

The data that support the findings of this guideline are publicly available, as referenced.
