# Supplementary material for: HLA genotype testing for carbamazepine, oxcarbazepine and eslicarbazepine: A guideline developed by the UK Centre of Excellence in Regulatory Science and Innovation in Pharmacogenomics (CERSI‐PGx)
Source: Br J Clin Pharmacol. 2026 Apr 19;92(7):1957–76. doi: 10.1002/bcp.70559 (PMC13304249; doi:10.1002/bcp.70559)
Supplement: Supplementary file 1 — Table S1. Competence, affiliations and disclosure of conflicts of interest of the writing committee of the CERSI‐PGx Guideline for HLA genotype testing for carbamazepine, oxcarbazepine and eslicarbazepine. Table S2. Comments received during the consultation period by various organ on the CERSI‐PGx Guideline for HLA genotype testing for carbamazepine, oxcarbazepine and eslicarbazepine and responses by the UK CERSI PGx writing committee. [file BCP-92-1957-s001.docx]

***HLA*genotype testing for Carbamazepine, Oxcarbazepine, and Eslicarbazepine: a guideline developed by the UK Centre of Excellence in Regulatory Science and Innovation in Pharmacogenomics (CERSI-PGx)**

Lucy Galloway^1,2,^*, Cinzia Dello Russo^3,4,^*, Nicholas Bass^5^, Elvira Bramon^5,6^, Helen Cross^7,8^, Natalie Curley^9^, Sarah Curran^10^, Helen Davies^11^, Jana De Villiers^12^, William Evans^13,14,15^, Bernhard Frank^16^, Alice Groves^17^, Judith Hayward^18,19,20^, Jon Higham^21^, Dyfrig A Hughes^22^, Shwe Sin Kyaw^22^, Anthony G Marson^3,16^, Ailsa McLellan^23^, Seth Mensah^24^, Francis O’Neill^25^, Jane Sargison^26^, Sanjay M Sisodiya^27,28^, Jill Swan^29^, Joanna M Zakrewska^30^, Munir Pirmohamed^3,31^.

*Lucy Galloway and Cinzia Dello Russo should be considered joint first authors.

^1^NHS South East Genomic Medicine Service, St George’s University Hospitals NHS Foundation Trust, London, UK.

^2^ Faculty of Life Sciences & Medicine, King’s College London, London, UK.

^3^Department of Pharmacology and Therapeutics, Institute of Systems, Molecular and Integrative Biology, University of Liverpool, Liverpool, UK.

^4^Department of Translational Medicine and Surgery, Section of Pharmacology, Università Cattolica del Sacro Cuore - Fondazione Policlinico Universitario A. Gemelli, IRCCS, Rome, Italy.

^5^Division of Psychiatry, University College London, London, UK.

^6^North London NHS Foundation Trust, London, UK.

^7^University College London, Great Ormond Street Institute of Child Health, London, UK.

^8^Great Ormond Street Hospital for Children NHS Foundation Trust, London, UK.

^9^NHS South West London Integrated Care Board, London, UK.

^10^South London and Maudsley NHS Foundation Trust, London, UK.

^11^Cwm Taf Morgannwg University Health Board, Abercynon, UK.

^12^The State Hospitals Boards for Scotland, Lanark, UK.

^13^Yorkshire Regional Genetics Service, Leeds, UK.

^14^Gibson Lane Practice, Leeds, UK.

^15^School of Medicine, University of Nottingham, Nottingham, UK.

^16^The Walton Centre NHS Foundation Trust, Liverpool, UK.

^17^Aneurin Bevan University Health Board, Urgent Care Division, Newport, UK.

^18^National Genomics Education Programme, NHS England, London, UK.

^19^NHS North West Genomic Medicine Service, Manchester, UK.

^20^Shipley Medical Practice, Affinity Care, Shipley, UK.

^21^Birmingham Community Healthcare NHS Foundation Trust and Birmingham Dental Hospital, Birmingham, UK.

^22^Centre for Health Economics and Medicines Evaluation, North Wales Medical School, Bangor University, Bangor, UK.

^23^Royal Hospital for Children and Young People, Edinburgh, UK.

^24^Cardiff and Vale University Health Board, Cardiff, UK.

^25^ School of Dentistry, Institute of Life Course and Medical Sciences, University of Liverpool, Liverpool, UK

^26^Faculty of Science and Engineering, Manchester Metropolitan University, Manchester, UK.

^27^Department of Epilepsy, UCL Queen Square Institute of Neurology, University College London, London, UK.

^28^ National Hospital for Neurology and Neurosurgery and Chalfont Centre for Epilepsy, University College London Hospitals NHS Foundation Trust, London, UK.

^29^NHS Ayrshire and Arran, Ayr, UK.

^30^Royal National ENT & Eastman Dental Hospitals, University College London Hospitals NHS Foundation Trust, London, UK.

^31^The Wolfson Centre for Personalised Medicine, Centre for Drug Safety Science, University of Liverpool, Liverpool, UK.

**Supplementary Methodology**

**Writing committee**

The UK CERSI-PGx guideline on *HLA* genotype testing for carbamazepine, oxcarbazepine and eslicarbazepine was developed by a multidisciplinary team, including experts in clinical pharmacology, pharmacology and pharmacogenomics; clinicians from the following disciplines: adult and paediatric neurology, facial pain, pain, oral surgery, oral medicine, adult and paediatric psychiatry, neuropsychiatry and primary care; and experts in pharmacy, genomic medicine and health economics. The guideline committee included five members from Scotland and three from Wales in addition to members from England. Full disclosure of the affiliations, competences and conflict of interests is provided in a separate document in the supplement. A preliminary meeting between LG and CDR was held on the 01 of July 2025 to develop a first draft of the guideline. The writing committee convened the first time online on the 05 of August 2025 and subsequently met online twice (on 28 August and on 06 October) to finalise the guideline draft for consultation, with written contributions from members throughout this period. Details on the writing committee are provided in Supplementary Table 1.

**Standardised template for the guideline**

The guideline is written according to a standard template developed previously by CERSI-PGx for their guidelines^1^.

**Literature review and prescribing recommendations**

Recommendations from the UK CERSI-PGx consortium are evidence-based together with expert input from committee members who were able to highlight nuances in specific clinical settings in which the drugs are used. A comprehensive literature review was conducted on the three main *HLA* alleles associated with increased risk of severe cutaneous adverse reactions in response to carbamazepine and related compounds, integrating information provided by the latest clinical pharmacogenetic guidelines developed by the Dutch Pharmacogenetics Working Group (DPWG)^2^, the Clinical Pharmacogenetics Implementation Consortium (CPIC)^3^, The Canadian Pharmacogenomics Network for Drug Safety (CPNDS)^4^, and more recently by the French National Network of Pharmacogenetics^5^. In the Evidence Overview section, a summary of the most relevant evidence is provided. An extensive literature review was performed on specific topics, including the association between *HLA-B*15:02* and *HLA-A*31:01* with other aromatic anti-epileptic agents that may be considered as potential alternatives to carbamazepine and related compounds; the association between *HLA-B**15:02 and lacosamide induced cutaneous toxicity; and in relation to the cost-effectiveness of genotyping, prioritising studies relevant to the UK NHS when available. The section on clinical actions based on pharmacogenetic test results contains prescribing recommendations on alternative drugs that need to be avoided because of the potential risk of toxicity. For alternative treatments options we refer the user to national and local guidelines. Whenever possible relevant UK prescribing guidelines have been quoted.

**Genetic variants to be tested**

The UK CERSI-PGx guideline recommends genotyping of three different *HLA* alleles, namely *HLA-B*15:02*, *HLA-B*15:11* and *HLA-A*31:01*. For these alleles the HGVS nomenclature available in the IPD-IMGT/HLA database (<https://www.ebi.ac.uk/ipd/imgt/hla/>) has been reported.

The allelic frequencies reported in Table 1 have been extracted from the HLA Allele Frequency Net Database^6^  according to the following search strategy:

***African (Sub-Saharan)***: **Region** Sub-Saharan Africa AND **Ethnic Origin** Black

***African American/Afro-Caribbean***: **Region** North America AND **Ethnic Origin** Black

***Asian (South-East)***: **Region** South-East Asia AND **Ethnic Origin** All ethnicity

***Asian (South)***: **Region** South Asia AND **Ethnic Origin** All ethnicity

***European***: **Region** Europe AND **Ethnic Origin** Caucasoid – For the *HLA-B*15:02*, this outcome includes also data related to the Spain, Andalusia, Romani population (99 samples) with allelic frequency of 1%.

***Latino***: **Region** All regions AND **Ethnic Origin** Hispanic – These outcomes include mostly Hispanic people living in the USA.

***Oceanian***: **Region** Oceania AND **Ethnic Origin** All ethnicity - For the *HLA-B*15:02*, this outcome includes also data related to the Philippines, Ivatan population (50 samples) with allelic frequency of 22%. For the *HLA-A*31:01*, this outcome includes also data related to the USA, Hawaii, Okinawa population (106 samples) with allelic frequency of 10.5%.

**Consultation process**

At the end of the writing process, the guideline final draft was shared in confidence for consultation with the Regulators (the MHRA, The National Institute for Health and Care Excellence, National Pharmacogenomics Group - NHS Wales), Specialty Societies relevant to the prescription of carbamazepine and related compounds, Pharmacy Professional Networks, the ClinPGx (CPIC) and the DPWG, Genomic Societies, like the Association for Molecular Pathology, the Association for Clinical Genomic Science, the British Society of Histocompatibility and Immunogenetics (BSHI) joint with the Welsh Blood Service and the NHS England Genomics Unit. In addition, the guideline was sent out for consultation to the Association of the British Pharmaceutical Industry, The British in vitro Diagnostics Association, and the UK-PGx Industry Network. The consultation period started on the 4 of December 2026 and was open until the 9 of January 2026, with a few weeks extension to collect input from most stakeholders. Comments received are included in a single table together with the UK CERSI-PGx responses to each of the comments, and if appropriate, by changes in the guideline. The content of the table is published as Supplementary Table 2 to ensure transparency of the procedures undertaken.

**Patient And Public Involvement and Engagement (PPIE)**

The guideline was shared with two patient representatives, members of the PPIE group established for the delivery of work package 5 of the UK CERSI-PGx project^7^. Patients participated in an *ad-hoc* meeting held on the 24 of November 2025. A lay summary has been prepared with their contribution to explain the relevance of *HLA* genetic variability on the benefits/risks profile of carbamazepine and related drugs. The lay summary also highlights the impact of genotyping on drug prescription. The lay summary is included in the supplementary material and will be included in the UK CERSI-PGx website (<https://cersi-pgx.org/>).

**Supplementary References**

1. Dello Russo C et al. CYP2C19 genotype testing for clopidogrel: A guideline developed by the UK Centre of Excellence in Regulatory Science and Innovation in Pharmacogenomics (CERSI-PGx). *Br J Clin Pharmacol*. 2026; 92: 329-347. doi: 10.1002/bcp.70370..
2. Manson LEN et al. Dutch Pharmacogenetics Working Group (DPWG) guideline for the gene-drug interaction of CYP2C9, HLA-A and HLA-B with anti-epileptic drugs. *Eur J Hum Genet*. 2024; 32: 903-911. doi: 10.1038/s41431-024-01572-4.
3. Phillips EJ et al. Clinical Pharmacogenetics Implementation Consortium Guideline for HLA Genotype and Use of Carbamazepine and Oxcarbazepine: 2017 Update. *Clin Pharmacol Ther*. 2018; 103: 574-581. doi: 10.1002/cpt.1004.
4. Amstutz U et al. Recommendations for HLA‐B15:02 and HLA‐A31:01 genetic testing to reduce the risk of carbamazepine‐induced hypersensitivity reactions. *Epilepsia*. 2014; 55:496–506. doi: 10.1111/epi.12564.
5. Renac V et al. Guidelines From the French-Speaking Society for Histocompatibility and Immunogenetics (SFHI) for Harmonisation of HLA Genotyping in Autoimmune Diseases, Drug Hypersensitivity and Pharmacogenetics. *HLA*. 2025; 106: e70442. doi: 10.1111/tan.70442.
6. Gonzalez-Galarza FF et al. Allele frequency net database.  Methods Mol Biol. 2024;2809:19-36.  <https://www.allelefrequencies.net/hla.asp>. Updated 2020. Accessed 24th November 2025.
7. Pirmohamed M, Dello Russo C. UK Centre of Excellence in Regulatory Science and Innovation in Pharmacogenomics. *Br J Clin Pharmacol*. 2026; 92(2):327-328. doi: 10.1002/bcp.70363.

**Supplementary Plain English Summary**

Kamil Sterniczuk and Abdur Rehman Hussain, members of the UK CERSI-PGx PPIE group.

**What is carbamazepine?**

Carbamazepine is a medicine used to treat epilepsy (seizures). It is also prescribed for trigeminal neuralgia, a condition that causes sudden, severe facial pain and sometimes for bipolar disorder, which affects mood. Carbamazepine works by calming overactive electrical signals in the brain. There are similar medicines called oxcarbazepine and eslicarbazepine, which are mainly used for epilepsy.

**Why do genes matter with carbamazepine?**

In some people, carbamazepine can cause serious allergic reactions, especially affecting the skin. These reactions are not dependent on the dose and are unpredictable. Research has shown that genes involved in the immune system, called *HLA* genes, can greatly increase the risk of these reactions.

People inherit different versions of these genes, which means that carbamazepine is safe for many people but can be dangerous for others.

The most important versions of the *HLA* genes associated with allergic reactions to carbamazepine are:

- *HLA-B*15:02*
- *HLA-B*15:11*
- *HLA-A*31:01*

Allergic reactions can also occur with oxcarbazepine and eslicarbazepine, albeit less frequently, often in association with the versions of the *HLA* genes reported above.

**What kinds of reactions can occur?**

Most reactions happen within the first three months of treatment, as follows:

- Mild reactions may include a skin rash.
- Severe reactions are rare but can be life-threatening and include:
  - Stevens–Johnson syndrome (SJS) and toxic epidermal necrolysis (TEN), where the skin blisters and peels.
  - DRESS syndrome, which can cause rash, fever, swollen glands, facial swelling, and damage to organs such as the liver or kidneys.

It is extremely important to prevent these adverse reactions.

**Who is at higher risk?**

- *HLA-B*15:02* and *HLA-B*15:11* are more common in people of Asian ancestry, but they can occur in anyone.
- *HLA-A*31:01* is more common in many populations including White British and European populations.
- People may not always know or report their ancestry, so relying only on self-reported ethnicity is not the best approach.

**What do the guidelines say?**

The UK guideline recommends genetic testing before starting carbamazepine, oxcarbazepine or eslicarbazepine in:

- Anyone who has never taken these medicines before
- Anyone who has taken them for less than three months

This applies to all patients, regardless of ancestry or reason for treatment.

**How do test results affect treatment?**

- If *HLA-B*15:02* is present, carbamazepine, oxcarbazepine, and eslicarbazepine should be avoided.
- If *HLA-A*31:01* is present and *HLA-B*15:02* is absent, these medicines should be avoided if an alternative is available.
- If *HLA-B*15:11* is present, these medicines should also be avoided if an alternative is available.

If none of these genetic risk factors are found, carbamazepine (or oxcarbazepine or eslicarbazepine) can usually be prescribed with greater confidence.

**What does this mean for patients?**

Genetic testing helps doctors choose the safest medicine before treatment begins. This reduces the risk of severe skin reactions and avoids harm that cannot be predicted by routine blood tests.

Test results should be saved in medical records so that all healthcare professionals are aware. Patients should be informed:

- why the test is done
- what the results mean
- how it affects their treatment options

**Regulatory considerations**

Carbamazepine product information already includes warnings about genetic risk.

The current guideline emphasises that testing should be accurate, available quickly and affordable so the results can guide treatment decisions without unnecessary delay.

**Research recommendations**

More research is still needed to understand:

- the impact of genetic variability on oxcarbazepine and eslicarbazepine safety
- the impact of genetic variability on other adverse reactions to these drugs and to other medicines with a similar chemistry
- The long-term cost-effectiveness of routine testing

**Key message**

Carbamazepine is an effective medicine but, in some people, it can cause rare and serious immune reactions. A simple genetic test can identify those at risk before treatment starts. When risk is found, alternative drugs are available. This approach improves patient safety and supports more personalised, informed care.

**Supplementary Table 1.** Competence, affiliations, and disclosure of conflicts of interest of the writing committee of the CERSI-PGx Guideline for HLA genotype testing for Carbamazepine, Oxcarbazepine, and Eslicarbazepine

| **Name** | **Main Expertise** | **Full Affiliation** | **Institution/s** | **Conflicts** | **Nation** |
| --- | --- | --- | --- | --- | --- |
| **Munir Pirmohamed**  **CHAIR** | Clinical Pharmacology Internal Medicine  Pharmacogenomics | Department of Pharmacology and Therapeutics, Wolfson Centre for Personalised Medicine, University of Liverpool, Liverpool, UK.  Liverpool University Hospital Foundation NHS Trust, Liverpool, UK. | University of Liverpool. | Currently receives partnership funding, paid to the University of Liverpool, for the MRC Medicines Development Fellowship Scheme (co-funded by MRC and GSK, AZ, Optum and Hammersmith Medicines Research). He has developed an HLA genotyping panel with MC Diagnostics but does not benefit financially from this. He is part of the IMI Consortium ARDAT ([www.ardat.org](http://www.ardat.org)); none of these of funding sources have been used for the guideline. | England. |
| **Lucy Galloway**  **RAPPORTEUR** | Pharmacy  Genomic Medicine Pharmacogenomics | Consultant Pharmacist Genomic Medicine, NHS South-East Genomic Medicine Service, St George’s University Hospitals NHS Foundation Trust, London, UK.  Honorary Senior Lecturer, King’s College London, London, UK. | NHS South-East Genomic Medicine Service.  King’s College London. | Genomics committee member for the UK Clinical Pharmacy Association, and member of NHS England Pharmacogenomics Test Evaluation Working Group. | England. |
| **Cinzia Dello Russo** | Pharmacology  Pharmacogenomics | Department of Pharmacology and Therapeutics, Institute of Systems Molecular and Integrative Biology (ISMIB), University of Liverpool, Liverpool, UK.  Department of Translational Medicine and Surgery, Section of Pharmacology, Università Cattolica del Sacro Cuore, Rome, Italy. | University of Liverpool.  Università Cattolica del Sacro Cuore. | No conflicts. | England. |
| **Elvira Bramon** | Psychiatry (adults)  Professor of Neuroscience and Mental Health | Faculty of Brain Sciences,  University College London, London, UK. | University College London.  North London Mental Health Partnership. | No conflicts declared. | England. |
| **Jana De Villiers** | Consultant Psychiatrist | High Secure Intellectual Disability Service for Scotland and N Ireland.  Forensic Network Clinical Lead for Intellectual Disability and Autism  The State Hospitals Board for Scotland, Lanark, UK. | NHS State Hospitals Board for Scotland. | No conflicts. | Scotland. |
| **Nicholas Bass** | Psychiatry | Clinical Associate Professor  Mental Health Neuroscience  University College London, London, UK. | University College London. | No conflicts. | England. |
| **Sarah Curran** | Paediatric Psychiatrist | Consultant Liaison Child and Adolescent Psychiatrist  National and Specialist CAMHS,  South London and Maudsley NHS Foundation Trust, London, UK. | South London and Maudsley NHS Foundation Trust. | No conflicts. | England. |
| **Jane Sarginson** | Pharmacogenetics  Stratified Medicine | Manchester Metropolitan University, Manchester, UK. | Manchester Metropolitan University. | No conflicts declared. | England. |
| **Sanjay M Sisodiya** | Professor of Neurology | Institute Deputy Director for Sustainability & Climate Change and Consultant Neurologist  Department of Epilepsy, UCL Queen Square Institute of Neurology, London, UK.    National Hospital for Neurology and Neurosurgery and Chalfont Centre for Epilepsy, London, UK.  Transformation Director, Epilepsy Society. | University College London.  National Hospital for Neurology and Neurosurgery and Chalfont Centre for Epilepsy. | Has received honoraria for educational events or advisory boards from Jazz Pharma, Angelini Pharma, Biocodex, Eisai, Zogenix/UCB and institutional contributions for advisory boards, educational events or consultancy work from Eisai, Jazz/GW Pharma, Servier, Stoke Therapeutics, Takeda, UCB and Zogenix. | England. |
| **Anthony G Marson** | Professor of Neurology | Professor of Neurology / Dean of the Institute of Systems, Molecular and Integrative Biology  Department of Pharmacology and Therapeutics  University of Liverpool, Liverpool, UK. | University of Liverpool.  The Walton Centre NHS Foundation Trust. | Has received grant income from Angelini and has undertaken consultancy for Jazz Pharma, Angelini, UCB Pharma, Alturix, all paid to University of Liverpool. | England. |
| **Joanna M Zakrzewska** | Consultant Facial Pain | Royal National ENT & Eastman Dental Hospitals, Pain Management Centre, National Hospital for Neurology & Neurosurgery, University College London Hospitals NHS Foundation Trust, London, UK. | Royal National ENT & Eastman Dental Hospitals. | No conflicts. | England. |
| **Francis O’Neill** | Oral Surgery | Senior Lecturer, Honorary Consultant in Oral Surgery, School of Dentistry  Institute of Life Course and Medical Sciences  University of Liverpool, Liverpool, UK. | University of Liverpool. | Serves on the Medical Advisory *Board for the Trigeminal Neuralgia Association.* | England. |
| **Jon Higham** | Consultant in Oral Medicine and Chief Clinical Information Officer | Birmingham Community Healthcare NHS Foundation Trust and Birmingham Dental Hospital, Birmingham, UK. | Birmingham Community Healthcare NHS Foundation Trust and Birmingham Dental Hospital. | No conflicts declared. | England. |
| **Dyfrig Hughes** | Health Economics  Pharmacy  Pharmacogenomics | Co-Director, Centre for Health Economics & Medicines Evaluation \| Cyd-Gyfarwyddwr, Canolfan Economeg Iechyd a Gwerthuso Meddyginiaethau North Wales Medical School, Bangor University, Bangor, UK. | Bangor University. | Dyfrig Hughes is Chair of NHS Wales National Pharmacogenomics Group, and co-chair of NHS England Pharmacogenomics Test Evaluation Working Group. | Wales. |
| **Judith Hayward** | General Practice | GP, Shipley Medical Practice, Affinity Care, Shipley, UK.  GPwER in Clinical Genetics/Genomics, Leeds Clinical Genomics Service.  Innovation Director, North West Genomic Medicine Service Alliance.  Primary Care Lead, National Genomics Education Programme, NHS England.  Royal College of General Practice Joint Clinical Representative in Genomic Medicine. | Shipley Medical Practice, Affinity Care.  National Genomics Education Programme, NHS England.  NHS North West Genomic Medicine Service, Manchester, UK. | No conflicts. | England. |
| **William Evans** | General Practice | GP, Gibson lane practice, Leeds, UK.  GPwER in Clinical Genetics/ Genomics, Yorkshire Regional Genetics Service.  Primary Care Lead for the North East and Yorkshire Genomic Medicine Service Alliance.    Honorary Assistant Professor,  School of Medicine, University of Nottingham. | Gibson Lane Practice.  Yorkshire Regional Genetics Service.  School of Medicine, University of Nottingham. | No conflicts. | England. |
| **Natalie Curley** | Pharmacy | Lead Pharmacist (Quality), Medicines Optimisation (Medicines & Safety),  NHS South West London Integrated Care Board, London, UK. | NHS South West London. | No conflicts. | England. |
| **Helen Davies** | Pharmacy | Principal Pharmacist in community and primary care, Cwm Taf Morgannwg University Health Board, Abercynon, UK. | Cwm Taf Morgannwg University Health Board. | Elected Welsh Board member of the RPS, and I am the Cwm Taf Morgannwg University Health Board representative on the All Wales Prescribing Advisory Group (part of AWTTC). | Wales. |
| **Seth Mensah** | Neuropsychiatry | NHS Wales  University Hospital of Wales, Cardiff and Vale University Health Board, Cardiff, UK. | University Hospital of Wales. | No Conflicts. | Wales. |
| **Shwe Sin Kyaw** | Health Economics | Post-doctoral health economics researcher,  North Wales Medical School,  Bangor University, Bangor, UK. | Bangor University. | No Conflicts. | Wales. |
| **Alice Groves** | General Practice | Aneurin Bevan University Health Board, Urgent Care Division, Newport, UK. | Aneurin Bevan University Health Board. | No conflicts declared. | Wales. |
| **Ailsa McLellan** | Paediatric Neurology | Consultant Paediatric Neurologist,  Royal Hospital for Children and Young People, Edinburgh, UK. | Royal Hospital for Children and Young People. | Has received honoraria for educational events, consultancy work or advisory boards from Jazz Pharma and Biocodex, and institutional contributions educational events from Jazz/GW Pharma and UCB. | Scotland. |
| **Helen Cross** | Paediatric Neurology | University College London, London, UK.  UCL- Great Ormond Street Institute of Child Health, London, UK. | University College London.  Great Ormond Street Institute of Child Health. | No conflicts declared. | England. |
| **Jill Swan** | Pharmacist | Principal Pharmacist Clinical Services  NHS Ayrshire and Arran, Ayr, UK. | NHS Ayrshire and Arran. | No conflicts. | Scotland. |
| **Bernhard Frank** | Consultant Pain Physician | Clinical Lead for Neuropathic Pain,  Research Lead for Pain Medicine at the Walton Centre, Liverpool, UK. | The Walton Centre  NHS Foundation Trust.  The University of Liverpool. | No Conflicts. | England. |

**Supplementary Table 2.** Comments received during the consultation period by various organizations on the CERSI-PGx Guideline for *HLA* genotype testing for Carbamazepine, Oxcarbazepine, and Eslicarbazepine and Responses by the UK CERSI PGx writing committee

| **Organisation** | **Comments from organisation** | **Responses from Committee** | **Changes to the Guideline (if any)** |
| --- | --- | --- | --- |
| **The Medicines & Healthcare products Regulatory Agency (MHRA)** | The authors have provided a comprehensive assessment of the evidence base underlying the association between HLA allele variants and hypersensitivity reactions following treatment with Carbamazepine, Oxcarbazepine, and Eslicarbazepine. The guideline provides a clear and justified stepwise approach for prescribing options to balance an individual patient’s benefit / risk profile according to their genetic background. The recommendations for pharmacogenomic testing are well justified and are aligned with international prescribing guidelines based on genotype.  Minor suggestion:  P10 – “MHRA safety measures and precautionary advice should be followed for sodium valproate” – cite reference 15 as done for this recommendation on p12 | Thank you for your support.  We amended the text according to your suggestion. | No.  Section 7.1. |
| **National Institute of for Health and Care Excellence (NICE)** | Section 5.1: Please could the first paragraph be amended to reflect the recommendations of NG127. NICE have a recommendation highlighting that carbamazepine and oxcarbazepine may exacerbate seizures in people with absence or myoclonic seizures, including in juvenile myoclonic epilepsy. | We amended the text according to your suggestion to include the statement “Carbamazepine and oxcarbazepine may exacerbate seizures in people with absence or myoclonic seizures, including juvenile myoclonic epilepsy and are not recommended for these seizure types”. | Section 5.1. |
| **National Pharmacogenomics Group (NHS Wales)** | No response received |  |  |
| **British Pharmacological Society (BPS)** | Not crucial, but language is important - "For example, the allelic frequency of HLA-B*15:02 is highest in Southeast Asian (0-36%) and South Asian (0-14%) populations (Table 1). However, this allele is not found at such high frequencies in all East Asian subpopulations, for example the frequency is low in Japanese (<1%)." - as it currently reads, I interpret it as saying that Southeast Asian and South Asian is a subpopulation of East Asian. I suggest "such high frequencies in all Asian subpopulations"  Also not crucial - Please consider if paragraphs 1+2 of page 2 should be moved higher up, for example after, paragraph 2 of Page 1. I would find it easier at reading about the drugs and adverse effects first then flowing onto the HLA biomarkers.  I like the idea for pre-emptive genotyping where not first-line treatment. [Important to clarify] However I don't think the health economics works out - My limited understanding of the pharmacoeconomic analysis is it is based on that testing is done for all patients who are about to be prescribed carbamazepine (and not pre-emptive testing). My expectation is that with pre-emptive testing, a significant proportion (and maybe even majority) will not need a second line drug. This probably shifts the analysis to not cost-effective (?).  As a non-neurologist or psychiatrist, I found discussion around aromatic agents difficult. Perhaps it's easier for a specialist in this area. May I suggest there can be a separate paragraph early in the main text highlighting the relationship between them and the potential increased risk.  It also would have helped me with a list/table of which drugs are in this group (rather than being hidden in block of text)  It is also unclear to me why lamotrigine appears to be "caution" whereas while other aromatic agents are "avoid if possible".  Few points on language.  You have used ‘patients’ rather than ‘people’. This may be an active style choice and sorry if I missed it in the last guide but just flagging that NICE use ‘people’ more frequently than ‘patients’.    Section 4.1: could I suggest is amended to ‘People/patients who have no or less than three months of exposure to ………’ (i.e. put the time period first)  In most places you use ‘pharmacogenetic’ as opposed to solely ‘genetic’ e.g. pharmacogenetic testing. But in others you drop the ‘pharmaco’. E.g. 'Actions based on genotype’ in title for section 7 with a follow up of ‘pharmacogenetic test results’. I would stick to one style. Title for section 7 could probably just be ‘Prescribing following pharmacogenetic testing’  Section 11 is really important (as a general section) but I am not sure the title of it indicates what it contains. If I were a casual user, I might not know to look there for drugs that may be co-prescribed and have issues. Could I recommend you consider a different title for this section for example ‘Considerations for other drugs that may be co-prescribed or considered in these populations’. I would also make the point in the opening of this section that there may be other co-prescribed medications for other indications that the section will not cover and clinicians should check for pharmacogenetics-based guidance for these drugs elsewhere.  The reviewer below mentions the language around ethnicity. I am not a good copy editor, but I wonder if you need someone to have a look through the text for how you have used language as there are areas that I am not certain the wording is quite right. For example, in para 1 of section 11 you state ‘evidence was mainly generated *in Asians’.*This language is not particularly person-centred (e.g. instead ‘among Asian participants/populations), the preposition is inaccurate (data are observed in or collected from rather than being *generated* in people) and ‘Asian’ covers around 60% of the global population so you might just say ‘people’! | We amended the text according to your suggestion.  We amended the text according to your suggestion.  Thank you for your comment. Our recommendation is that any treatment naïve patient who is about to be prescribed carbamazepine, oxcarbazepine, or eslicarbazepine should undergo pharmacogenetic testing. We have amended the text according to your comment, to remove reference to pre-emptive testing. This has now been changed to “In patients in whom alternative treatments are unsuccessful or contra-indicated, testing should be requested as soon as possible if treatment with carbamazepine or related compounds is considered likely in the future.”  Thank you for your comment. We have included a paragraph at the end of Section 3.  We have now reported the list of other aromatic antiepileptic drugs at the end of Section 3. Recommendations regarding which alternative treatments should also be avoided is included in Tables 3-5 which summarise the recommended clinical actions based on pharmacogenetic test results in epilepsy, bipolar disorder, and trigeminal neuralgia.  We have amended the recommended clinical actions based on pharmacogenetic test results in epilepsy, bipolar disorder, and trigeminal neuralgia in Sections 7.1, 7.2, 7.3 and Tables 3-5 to align with the recommendation in Section 11.2 that, through an abundance of caution, the CERSI-PGx recommend the avoidance of lamotrigine unless the benefit exceeds risk, and an alternative agent is not available.  Thankyou, we have reviewed the style choice, and we have used “patients” when we are referring to all patients/people with any condition/indication, but where we are referring to one specific condition (for example in section 7) we have used “person living with X condition”. Please note the following reference **Costa DSJ et al. Patient, client, consumer, survivor or other alternatives? A scoping review of preferred terms for labelling individuals who access healthcare across settings. BMJ Open 2019;9:e025166. doi:10.1136/ bmjopen-2018-025166.**  The text has been amended in both Section 4.1 and 4.2 as suggested  Thank you for the suggestions, we harmonized the wording across the guideline, using “pharmacogenetic testing” in place of “genetic testing” or “genotyping”.  Thank you for your suggestion. We have included subheadings for this section to clearly highlight its content. We added a disclosure about other drugs, potentially co-prescribed with carbamazepine, whose pharmacogenetics is not covered in this section.  Thanks for this suggestion. We amended the text according to this (as well as previous) comment. | Section 1.  Section 1.  Section 5.1.  Section 3.  Section 3.  Sections 7.1, 7.2, 7.3. Tables 3,4,5.  All text.  Sections 4.1 and 4.2.  All text.  Section 11.  Section 11. |
| **Association of British Neurologists** | Our epilepsy advisory group commented that in current practice, carbamazepine is rarely initiated for epilepsy, and some clinicians cannot recall the last time they prescribed it in this context. Most clinicians already request HLA testing in high-risk ethnic groups, but testing in all patients is not currently standard practice. Carbamazepine is initiated much more commonly in trigeminal neuralgia, where is it first line and often the most effective treatment. Our advisory group did not agree that it would be appropriate to delay starting treatment for pharmacogenomic testing in trigeminal neuralgia, where very severe pain necessitates urgent treatment. | We agree with your comment. In section 5.3 Trigeminal neuralgia we have recommended “testing should normally be undertaken prior to prescription unless the clinical benefits clearly outweigh the risks”, and that “Based on a shared decision-making model, a patient and prescriber may collaboratively decide whether to proceed with treatment immediately or to wait for a result, considering the specific risks based on the patient’s ancestry”. In addition, section 5.5 provides recommendations for starting clinically urgent treatment before a test result is obtained. | No. |
| **Royal College of general Practitioners** | No response received |  |  |
| **UK Clinical Pharmacy Association (UKCPA) Genomics committee** | Overall, a very informative guideline that is easy to read and suitable for healthcare professionals with little prior experience of genomics. Given the numerous indications for carbamazepine, the guideline is quite lengthy, which may be unavoidable, but there is the risk that some of the key messages may become a little lost. | Thank you for your support. | No. |
|  | I attempted for due diligence to double check the HLA allele frequencies, but I was unable to do this using the cited database. How are these collated from the HLA database and converted into the geographical groups listed? We have also some suggestions for this in the feedback. | We have reported the detailed search strategy adopted in the methodology of the guidelines which is included as supplementary material so that analysis of the allelic frequencies can be replicated | Methodology, Supplementary data. |
|  | **Minor formatting/cosmetic comments:**   - Where mcg is written suggest writing in full as ‘microg’ or ‘microgram’ as per common practice - In 5.1 – ‘first line’ should be hyphenated. (As it is written in 5.2). 5.2 also has “fourth line” not hyphenated. 5.3 first line not hyphenated again. - Clean up reference 29 “&nbsp” in bibliography - Table 3 – consider reformatting so that if printed all footnotes sit on the same page as the table if relevant nearer to publication (irrelevant for online format) - Couple of typos here and there e.g. in section 12.1.   Layout: If possible, consideration may be given to having the summary of both evidence of clinical utility and cost effectiveness following each other? | Thank you for your comments, we have amended the text according to your suggestions.  We have maintained the structure to be consistent with other UK CERSI-PGx guidelines. | All text. |
|  | **Section 5 ‘incorporation into pathways’:**  Clarify what is meant by “testing before prescribing.” If the intended message is that carbamazepine should not be initiated until HLA results are available, this should be stated explicitly.  The phrase “availability of alternatives” could be interpreted variably; consider making the intended operational meaning clearer.  If the guideline anticipates prescribing an alternative while awaiting results, acknowledge that the alternative may be continued long term if effective, and this could influence both clinical utility and health economic modelling (i.e., switching behaviour and downstream outcomes).  If presenting a holistic view of factors beyond genotyping, consider explicitly including patient choice/preferences as a factor influencing treatment selection. | We have recommended “testing should normally be undertaken prior to prescription unless the clinical benefits clearly outweigh the risks.” In addition, section 5.5 provides recommendations for starting clinically urgent treatment before a test result is obtained.  We have stated that “At all stages of treatment (first-line, second line and add-on treatments), recommended alternatives to carbamazepine, oxcarbazepine and eslicarbazepine are available” and recommended that “Given the availability of alternative therapeutic options, testing should normally be undertaken prior to prescription unless the clinical benefits clearly outweigh the risks”. We have updated the text to specify “In patients in whom alternative treatments are unsuccessful or contra-indicated, testing should be requested as soon as possible if treatment with carbamazepine or related compounds is considered likely in the future.”  We have acknowledged that for indications other than trigeminal neuralgia, carbamazepine, oxcarbazepine and eslicarbazepine are no longer recommended as first line treatments and that at all stages of treatment (first-line, second-line and add-on treatments), recommended alternatives to carbamazepine, oxcarbazepine and eslicarbazepine are available”.  We have not been prescriptive regarding choices of alternatives and have signposted to relevant clinical guidelines from NICE and specialist societies which provide a holistic view of factors beyond genotyping including patient preferences as a factor influencing treatment selection. For trigeminal neuralgia, the only indication for which carbamazepine or a related compound is recommended as a first-line treatment, we have recommending that a patients and prescriber collaboratively decide on treatment options based on a shared decision-making model. | No.  Section 5.1.  No.  No. |
|  | **Patient counselling and actions in the event of rash (multiple sections)**  Current wording advises patients to report rash but does not explicitly state whether to stop carbamazepine or who to contact.  Consider strengthening and standardising the guidance throughout:   - advise patients to seek urgent medical advice immediately if rash occurs, - clarify whether they should withhold further doses pending clinical assessment (as locally appropriate), - state who to contact (e.g., GP/urgent care/ED/epilepsy team) and the urgency threshold. | Where we have advised reporting of skin rash, we have changed the text in Tables 3-5 to say “provide patients with advice on what to do if a skin rash occurs” and the following text in the table footnotes “It is common to get a skin rash with carbamazepine and related compounds. Most skin rashes are not serious. Patients should be advised to seek medical advice if a skin rash occurs and to go to A&E immediately if they experience a severe rash with flushing, blisters or ulcers as these can be signs of Stevens-Johnson syndrome”. The following reference for this advice is provided:  <https://www.nhs.uk/medicines/carbamazepine/side-effects-of-carbamazepine/>. | Abstract;  Sections 7.1, 7.2, 7.3; Tables 3,4,5. |
|  | **Clinical utility and health economic evaluation**  Integration and clarity of evidence claims:  The evidence review states clinical utility of HLA-B*15:02 has not been proven in European populations for SJS/TEN, likely due to low allele frequency. A cited analysis (reference 66) is described as indicating cost-effectiveness in European populations; further detail would be helpful, including:   - whether the analysis relates to panel testing vs single-gene testing, - what outcomes drove cost-effectiveness (e.g., SJS/TEN specifically or broader Type B ADRs), - how assumptions about alternative prescribing and switching behaviour were handled.   For these reasons, it may be beneficial to more tightly integrate the evidence review and health economic evaluation sections. | Further details of the economic evaluation referred to (Plumpton et al. doi: 10.1002/cpt.1312) have now been added, specifically that it: relates to a single gene test; considers ADRs besides SJS/TEN and hypersensitivity syndrome (i.e. maculopapular exanthema); and considered lamotrigine and valproate as the alternative anti-seizure medications. | Section 9. |
|  | **Allele frequency table (Table 1)**  Consider presenting allele frequencies as percentages rather than proportions (fraction of 1), to improve readability for non-experts and align with the style used in the final clopidogrel guideline and the rest of this guideline.  Address inconsistencies between narrative and Table 1: the text states HLA-A*31:01 ranges 0–7% in Asians, but Table 1 shows values up to 0.19 (19%); later text cites Japanese frequency as high as 17.5%.  A brief caveat/disclaimer may be needed noting that published frequencies vary across subpopulations and studies.   - Given the rarity of SJS/TEN, consider providing risk in an additional communication-friendly form (e.g., “1 in X”) in lower frequencies, to support patient discussions (as referenced in Section 5.3). - A “number needed to genotype” could be included, though may be less relevant in a comprehensive pre-emptive testing context. | We have changed the allele frequencies in Table 1 to percentages. We addressed inconsistencies between Table 1 and the text of Section 1. Allelic frequencies are derived from the HLA Allele Frequency Net Database.  A brief disclaimer is now included. We have added 1 in X probabilities to the incidence rates for severe cutaneous reactions to carbamazepine and oxcarbazepine in Section 1. The estimated NNT related to HLA-B*15:02 and HLA-A*31:01 testing is now reported in Section 3. | Table 1, Section 1.  Section 1, Section 3. |
|  | **Section 6: Genotyping method and reporting**  As the section discusses genotyping methods, consider adding a brief statement on HLA typing resolution and standardisation, e.g., minimum reporting at second-field resolution and handling of ambiguity (to be confirmed with a technical specialist/laboratory lead). | Noting also the comments received from ACGS we have amended the text to include a statement on minimum of two-field resolution and information on the appropriate laboratory accreditation standards (ISO 15189 and/or European Federation for Immunogenetics accreditation), | Section 6. |
|  | **SNOMED CT and structured reporting**  Table 2 provides SNOMED CT concepts for “detected” results but lacks structured codes or an interim strategy for “not detected/negative,” results.  While negative codes may be seen as lower informational value, they are important for:   - EHR clinical decision support (to permit safe prescribing), - transfer of care when testing occurred elsewhere, - avoiding unsafe assumptions if results are not recorded in structured format.   As noted in Section 5.6, if only positive results are communicated, a patient lost to follow-up could be incorrectly assumed to be negative. | We have added the codes to Table 2 as you suggested. | Table 2. |
| **UK Clinical Pharmacy Association (UKCPA) Neurosciences committee** | **General support and implementation realism:**   - We strongly support offering HLA testing prior to carbamazepine (and related compounds), given the severe burden, complexity of care, and mortality associated with TEN. - Carbamazepine is rarely used for acute seizure management, so there is usually time to test—provided results are available rapidly (ideally within a few days; certainly <7 days). - There is concern that testing is not currently widely available. The guideline recommendations may help create impetus for NHS laboratory provision as testing currently remains inconsistent and disparate across geographies, while facilitating the increase of testing capacity in the NHS. - We will need to make sure that does not create patients’ inequalities in accessing testing and therefore treatment. Previous lessons in tests made available through one lab or one commissioning pathway have introduced inequity and service inequality.   We are grateful for the opportunity to comment and hope these are helpful. We look forward to seeing the final updated guideline. | Thank you for your support.  Regarding the availability of testing, the logistics of the implementation is beyond the remit of this guideline. The UK CERSI-PGx focused on the clinical utility of pharmacogenetic testing and raising awareness around the increased risk of severe cutaneous adverse drug reaction in patients who carry relevant HLA gene variants. In this regard, we expect that the publication of the guideline may be a positive driver towards implementation.  Thank you again for your feedback and support with the guideline development. | No. |
|  | **Equity and access concerns**  Avoiding carbamazepine purely on the basis of Asian ethnicity is considered an overly crude approach that may compromise care where alternatives are limited.  Testing offers clinical certainty and avoids both over- and under-restriction.  Multiple committee members expressed concern that limited availability or single-lab pathways risk inequity and service inequality; equitable, routine access via local (or at least regional) NHS laboratories is strongly preferred. | Thank you for your comment which is aligned with the UK CERSI-PGx recommendations. The guideline underlines the limitations of offering HLA testing based only on ethnicity. Testing should be requested for all patients that require treatment with carbamazepine and analogues. As mentioned above the implementation of routine clinical testing is beyond the remit of the guideline. | No. |
|  | **Suggested additions to health economics (severity/cost burden):**   - Consider explicitly referencing the substantial burden of TEN including prolonged critical care admissions, often including intubation, high mortality risk, extreme pain and prolonged recovery.   This may strengthen the context for the health economic evaluation. | Details of the burden of SJS/TEN are presented in the Background/Overview section | No. |
|  | **Risk of replicating NICE lists of alternatives**  Concern that parts of the document replicate NICE guidance too specifically (e.g., extensive bullet lists of alternative medicines for seizures and bipolar disorder), which could become outdated if NICE guidance changes, or may distract from the PGx focus of the document. It may be better to focus more on simple alternative drug signposting rather than explicit lists and pathways, and provide a more general statement and direct readers to current NICE guidance for alternatives. Similarly, off-label status commentary for non-PGx-related medicines (e.g., fluoxetine/olanzapine/lamotrigine) may be unnecessary for this guideline’s scope. | We have referred to NICE guidelines in each section, and feel that the list of medications which are recommended by NICE should be mentioned in brief. | No. |
| **Royal Pharmaceutical Society** | No response received |  |  |
| **College of Mental Health Pharmacists** | Many thanks for asking us to contribute to this consultation.  We feel it is an excellent guideline with a comprehensive explanation of the risks and benefits of HLA genotyping prior to the use of carbamazepine and other similar drugs.    We agree with your description about how carbamazepine is used for psychiatric indications.  It is not a first line drug, and your description of alternative treatments according to NICE is accurate. It is not a comprehensive list of all medicines that we might consider using, but that is not the purpose of this guideline.   Anecdotally we have heard of increases in the use of carbamazepine for mood disorders following the latest MHRA restrictions on valproate, however we agree that there is no published evidence to confirm this.    We suggest that you should add 'alcohol withdrawal' to the list of other indications for carbamazepine in section 5.4, as it is currently recommended in UK clinical guidance for this indication. [Clinical guidelines for alcohol treatment - 10. Pharmacological interventions - Guidance - GOV.UK](https://www.gov.uk/guidance/clinical-guidelines-for-alcohol-treatment/10-pharmacological-interventions).    We note the lack of health economic data to support HLA genotyping prior to the use of these drugs in bipolar disorder and are supportive of the conclusion that further research in this area is required overall.    The only limitation that we would like to suggest, is that it is unclear how prescribers are expected to implement the guidance, if they do not have knowledge about how to access genetic testing for their patients.  At present we understand that HLA genotyping is not nationally funded in England, as it is not currently listed on the NHS genomic test directory.  You have alluded within the guidance (section 4.1) that the availability of genetic testing may vary according to geography.  It may be useful to signpost readers to the NHS genomic service, or equivalent in devolved nations, so that prescribers can correctly direct themselves to local testing services.    We look forward to seeing the publication of the final document. | Thank you for the positive comments and support.  Thank you for your support.  Thank you for your suggestion. We have included “medically assisted withdrawal from alcohol” in Section 5.4 as requested.  Thank you for your support on this issue.  The implementation strategy in the NHS is beyond the remit of this guideline. In this regard, we expect that the publication of the guideline may be a positive driver towards implementation. | No.  No.  Section 5.4.  No.  No. |
| **British and Irish Society for Oral Medicine** | No response received |  |  |
| **The Royal College of Surgeons (England)** | The RCS England Genomics and Molecular Surgery Group do not have any comments on the guidance. | Thank you for your response | No. |
| **British Pain Society** | Section 5.3 “Carbamazepine is routinely initiated in primary care and dentistry, but expert advice or referral to a specialist pain or condition-specific service”  Comment: In some regions, CBZ may require specialist initiation and not started routinely in primary care for TN.  Section 5.4 “Oxcarbazepine is used off-label for trigeminal neuralgia, and carbamazepine and oxcarbazepine are used off-label for other types of neuropathic pain”  Comment: See for most recent NeuP SIG recommendations.  Panel: Drugs or drug classes or neuromodulation treatments for neuropathic pain with inconclusive recommendations or recommendations against use based on the GRADE classification in **Soliman N et al. NeuPSIG Review Update Study Group. Pharmacotherapy and non-invasive neuromodulation for neuropathic pain: a systematic review and meta-analysis. Lancet Neurol. 2025 May;24(5):413-428. doi: 10.1016/S1474-4422(25)00068-7**.  Section 7.3 notes below Table 5 “Local guidance and MHRA safety measures and advice regarding the risk of abuse and dependence should be followed for gabapentin and pregabalin”  Comment: change “abuse” to “misuse”. | Thank you for your comments. In Section 2 we have stated that “the prescriber pool for carbamazepine includes (but not limited to) primary care, dentistry, neurology, neurosurgery, pain medicine, oral medicine and oral surgery”. We have also amended the text in Section 5.3 as follows to make this clearer: “Carbamazepine may be initiated by specialists or in primary care and dentistry. If initiated by non-specialists expert advice or referral to a specialist pain or condition-specific service is recommended if carbamazepine is not effective, not tolerated, or is contra-indicated.  Thank you, we have added this reference to the bibliography.  Thank you, we have amended the text as you suggested. | Section 5.3.  Section 5.4.  Sections 7.1, 7.3. |
| **Royal College of Psychiatrists** | Thanks for the opportunity to comment on this guideline, I am pleased to see that there were several psychiatrists and wider mental health professionals on the committee and that there was involvement from across UK nations. Firstly, I would like to commend the authors of the guideline and the committee for putting together such a well written, clear and systematically evidenced document.  I have only a few relatively minor points:  The main question I had was relating to the cost effectiveness of the proposal to genotype everyone (regardless of ethnicity) given the low frequency of the relevant alleles in those of European ancestries. Given the discussions for ACKR1 I also appreciate it is challenging to undertake anything other than generalised testing (regardless of ethnicity). Furthermore I am reassured about this concern by the UK NHS based modelling paper (ref 76) which incorporates whole population modelling of testing and is certainly supportive of the guideline recommendations.  A minor but important point is that on Table 6 for the CERSI column it does not mention that the recommendation is focussed on those who are treatment naive or <3/12 treatment. This is stipulated for the CPIC guideline in the table.  A related point is that there is very little mention of advice regarding those that may be prescribed carbamazepine on more than one occasion. Many psychiatric patients, including those with bipolar, will stop and restart medication frequently and often after short durations of adherence. To have some text and guidance regarding advice about HLA testing in this context would be good - it may be that if anyone has taken the medication in one ‘epoch’ for longer than 3 months they don’t need testing for instance but if there is no evidence to inform this then this should at least be recognised and mentioned.  Lastly, I wonder has there been any input or consultation with people with lived experience of epilepsy or bipolar as part of the guideline development - or is this planned? I think this would further strengthen the guidelines and would support its implementation, certainly within psychiatry and I think the patient/lived experience voice would be supportive and informative. | Thank you for your support.  Thank you for your comment and your support of our recommendation that all patients, regardless of ancestry, who are about to be prescribed carbamazepine, oxcarbazepine, or eslicarbazepine should undergo pharmacogenetic testing. Note also our recommendation to test for the following clinically relevant alleles: *HLA-B*15:02*, *HLA-B*15:11,* and *HLA-A*31:01*. Historically testing in the UK has been largely limited to the *HLA-B*15:02* allele which has a low allele frequency in European ancestries (0-1%). However, *HLA-A*31:01* has a frequency of up to 7% in Europeans.  The UK CERSI-PGx recommendation to avoid carbamazepine or related drugs is for all patients with a positive pharmacogenetic test result. Table 6 is a summary of therapeutic recommendations based on pharmacogenetic test results and does not include recommendations on who or when to test. UK CERSI-PGx recommend that testing is only indicated in patients who have no or less than 3 months exposure to carbamazepine or a related drug.  Our recommendation is that as well as treatment naïve patients, patients who have previously taken carbamazepine for less than 3 months should also undergo testing. This includes patients that have been on/off treatment.  We have had input from the patient advisory panel who work in WP5 within the CERSI.  They do not have lived experience of epilepsy but have lived experience of other conditions.  They have also written a lay summary for this guideline which is published alongside the main guideline. | No.  No.  No.  No. |
| **Bipolar UK** | No response received |  |  |
| **British Paediatric Neurology Association (BPNA)** | No response received |  |  |
| **The Association of the British Pharmaceutical Industry (ABPI)** | No response received |  |  |
| **The British In Vitro Diagnostic Association (BIVDA)** | No response received |  |  |
| **ClinPGx** | No response received |  |  |
| **The Dutch Pharmacogenetics Working Group (DPWG)** | No response received |  |  |
| **The Association for Clinical Genomic Science (ACGS)** | One of the main problems with star alleles is that their definition at the actual genotype level can change over time, and this could create real problems and confusion for diagnostic labs.  The ACGS has encouraged the use of HGVS nomenclature in clinical lab reports for several years now, through our best practice guidelines, and this has led to improvements in the consistency of reporting, as evidenced through external quality assessments by GenQA.  So I would urge you to include HGVS nomenclature in all your guidelines as standard, to help labs ensure they are all testing the same variants.  In section 4.1: “Pharmacogenetic testing may not be required if genetic information is already available in their medical record.”  This is a valid statement, if the results can be relied upon. If those results are from a diagnostic laboratory with ISO 15189 accreditation for the test, those results should be reliable.  However, results from non-accredited sources (e.g. some direct-to-consumer tests) might need further scrutiny before acting on those results.  The BSGM has published guidelines on direct-to-consumer testing and reliability of results, which could be referenced here.  We recommend a comment on use of existing genetic results should be a standard feature in all PGx guidelines going forward.  In section 6: “We recommend direct HLA typing laboratory methods rather than the use of tag-SNPs”  We recommend elaborating on both terms in this sentence for clarity.  (a) “Direct HLA typing laboratory methods” include antigen testing as well as genetic testing. We presume you are recommending genetic testing, and we suggest being more specific here.  (b) The term “tag-SNPs” is not one that is commonly used in diagnostic genomic laboratory and clinical settings. It is probably more commonly used in research and GWAS. Therefore, we recommend briefly outlining what is meant by tag-SNPs. | Thank you for your comment. The HGVS nomenclature has been included as requested.  Thank you, we have amended the text as follows to include your suggestions “We recommend exercising caution if presented with pharmacogenetic test results from direct-to-consumer or commercial providers that lack proper accreditation and robust external quality assurance procedures. Healthcare professionals are advised not to take results from non-accredited laboratories at face value.” We have added the suggested reference to the bibliography.  Thank you for confirming that the term “tag-SNPs” is not commonly used in diagnostic genomic laboratories. We have removed this sentence and replaced it with information on the appropriate laboratory accreditation standards (ISO 15189 and/or European Federation for Immunogenetics accreditation), and a recommendation that any ambiguities associated with the results should be reported. | Section 6.  Section 4.1.  Section 6. |
| **UK Industry Pharmacogenomics Network (UK-IPN)** | Section 1. Background and Overview: This is an excellent document, but it may be helpful to have a contents list. This would help people navigate directly to the appropriate recommendation table.  Section 4.1: “The availability of genetic testing”: Is this genetic testing for HLA-B*15:02, HLA-B*15:11 and HLA-A*31:01? If so, it would be helpful if this was clear. Thanks  Section 5.1 “In this case treatment can be initiated by specialists with testing undertaken at the same time” It would be useful to add here who would initiate the testing? I am assuming the specialist?  Section 5.2: “testing should be undertaken prior to prescription  unless the clinical benefits clearly outweigh the risks.” Is this genetic testing for HLA-B*15:02, HLA-B*15:11 and HLA-A*31:01? If so it would be useful to add to make this clear.  Section 5.3: “Testing should be undertaken prior to prescription unless the clinical benefits clearly outweigh the risks.” Is this genetic testing for HLA-B*15:02, HLA-B*15:11 and HLA-A*31:01? If so it would be useful to add to make this clear.  Section 5.4: “Testing should be undertaken prior to prescription unless…” Is this genetic testing for HLA-B*15:02, HLA-B*15:11 and HLA-A*31:01? If so it would be useful to add to make this clear.  Section 5.5: “there is still benefit in testing at the time of treatment initiation” Is this genetic testing for HLA-B*15:02, HLA-B*15:11 and HLA-A*31:01? If so it would be useful to add to make this clear.  Section 6: As SJS is discussed as allergic reaction/ adverse reaction, should these results be included in the red flag section on a patient notes with allergies, while waiting for electronic record card full integration?  Section 7.3: “Clinicians should also consult the relevant clinical guidelines from specialist societies, Royal Colleges, or NICE” have you considered live links for these guidelines to make sure users check guidelines | The guidelines will be formatted according to the Journal style, upon publication.  Thank you, we have amended the text according to your suggestion.  Thank you, we have amended the text according to your suggestion.  Thank you, we have amended the text according to your suggestion.  Thank you, we have amended the text according to your suggestion.  Thank you, we have amended the text according to your suggestion.  Thank you, we have amended the text according to your suggestion  The presence of a clinically relevant *HLA* allele does not guarantee an adverse drug reaction will occur but represents an increased risk. In their 2012 meta-analysis, Yip et al. estimated the positive predictive value for a *HLA-B*15:02* test to prevent carbamazepine-induced SJS/TEN was 1.8%. (**Yip VL et al. HLA genotype and carbamazepine-induced cutaneous adverse drug reactions: a systematic review. Clin Pharmacol Ther. 2012 Dec;92(6):757-65. doi: 10.1038/clpt.2012.189. Epub 2012 Nov 7. PMID: 23132554**.)  We are aware that, in the absence of agreed systems and an approach for recording of pharmacogenetic test results, some centres have utilised the allergy section of electronic health records for this purpose. However, if comprehensive and pre-emptive pharmacogenetic testing is adopted in the NHS as expected over the next 10 years, then this is unlikely to be an adequate mechanism for recording pharmacogenetic results due to the high number of drug-gene interactions likely to be relevant for an individual. We therefore recommend structured data capturing using relevant SNOMED-CT codes that will enable future interoperability between the electronic health record and pharmacogenetic Clinical Decision Support tools.  Patients who present with, or who have a history of drug reactions including cutaneous adverse drug reactions, must have these documented as a drug allergy in accordance with local and national guidelines (for example **NICE Clinical Guideline CG183 Drug allergy: diagnosis and management** Available at: <https://www.nice.org.uk/guidance/cg183/chapter/Key-priorities-for-implementation>)  Live link will be included in the reference list upon publication, as per journal policy | No.  Section 4.1.  Section 5.1.  Section 5.2.  Section 5.3.  Section 5.4.  Section 5.5.  No.  No. |
| **Association for Molecular Pathology**  **(AMP)** | The AMP is unable to undertake the revision during the time frame proposed. | Thank you for your response | No |
| **Faculty of Dental Surgery of the Royal College of Surgeons of England** | The Faculty of Dental Surgery of the Royal College of Surgeons of England does not have any comments on the guidance. | Thank you for your response | No |
| **British and Irish Society for Oral Medicine (BISOM)** | No response received |  |  |
| **Association of British Academic Oral & Maxillofacial Surgeons (ABAOMS)** | The draft guideline for the use of HLA genotype testing for Carbamazepine, Oxcarbazepine, and Esclicarbazepine seeks to provide a pathway of identifying patients at risk of developing recognised cutaneous side effects/eruptions and mitigating this risk for patients requiring these drugs for medical treatment of epilepsy, bipolar disorder, and trigeminal neuralgia.  The guideline has drawn upon available evidence in the literature as well as from other established international pharmacogenetics consortia to address this question.  The recommended testing for HLA-B*15:02, HLA-A*31:01 or HLA-B*15:11 alleles which are known to be associated with the drug cutaneous reactions is appropriate  The proposed pathways and timelines are acceptable and in line with the published evidence.  The draft guideline has taken into account the variations in individual responses and cautioned on importance of assessing each patient’s risk-benefit profile.  The guideline will help promote safe efficacious patient care. | Thank you for your support. | No |
| **Trigeminal Neuralgia Association UK (TNA UK)** | No response received |  |  |
| **Royal College of Anaesthetists**  **Faculty of Pain Medicine** | Thank you for the considerable work undertaken to produce the UK CERSI-PGx Carbamazepine Guideline. The document provides a rigorous synthesis of the genetic associations underpinning carbamazepine-induced adverse reactions and represents an important step toward personalised prescribing within the NHS. From the perspective of UK pain medicine, however, several areas of clarification and further development would strengthen the guideline’s applicability to our specialty and ensure proportionate, safe, and feasible implementation. | Thank you for your support. |  |
|  | **Science /Evidence Base and Relevance to Pain Medicine**  The guideline clearly presents strong genetic associations for HLA-B*15:02 and HLA-A*31:01, but almost all prospective validation originates from epilepsy cohorts, primarily in East and South East Asian populations. Evidence in neuropathic pain or trigeminal neuralgia populations is extremely limited. Given that carbamazepine is a cornerstone therapy in trigeminal neuralgia, it would be helpful for the guideline to:   - Acknowledge more explicitly the evidence gap for pain indications. - Discuss how transferable epilepsy-derived effect sizes and risk profiles are to pain populations. - Outline priority areas for UK research to generate pain-specific data. Highlight the role of penetrance and polygenic genetic risk influence on side effects [if any]. | The UK CERSI-PGx acknowledges the relevance of this observation. The text in Section 3 was amended highlighting the limited data available on the association between *HLA-B*15:02* and SJS/TEN in patients with trigeminal neuralgia (**Kulkantrakorn K et al. HLA-B*1502 strongly predicts carbamazepine-induced Stevens-Johnson syndrome and toxic epidermal necrolysis in Thai patients with neuropathic pain. Pain Pract. 2012 Mar;12(3):202-8. doi: 10.1111/j.1533-2500.2011.00479.x.Kulkantrakorn K et al., 2011).**  We have specified the relevance of the *HLA-B*15:02* association with carbamazepine induced SJS/TEN and the utility of the pharmacogenetic testing across the different indications (**Chen P et al. Carbamazepine-induced toxic effects and HLA-B*1502 screening in Taiwan. N Engl J Med. 2011 Mar 24;364(12):1126-33. doi: 10.1056/NEJMoa1009717**).  We have further included the need for additional research on the topics included in this comment in Section 12.  In terms of penetrance, we have reported odd ratios from original studies to enable comparison of the risk of carbamazepine-induced cutaneous ADRs in patients with and without clinically relevant HLA alleles. In their 2012 meta-analysis, Yip et al. estimated the positive predictive value for a *HLA-B*15:02* test to prevent carbamazepine-induced SJS/TEN was 1.8%. (**Yip VL et al. HLA genotype and carbamazepine-induced cutaneous adverse drug reactions: a systematic review. Clin Pharmacol Ther. 2012 Dec;92(6):757-65. doi: 10.1038/clpt.2012.189. Epub 2012 Nov 7. PMID: 23132554**). A quantifiable influence of polygenic risks on the risk of carbamazepine-induced cutaneous ADRs is not known, however we acknowledge that the genetic risk of an individual is not limited to a single gene but is likely to involve a complex interplay of HLA alleles and potentially other genetic and non-genetic factors. | Section 3.  Section 3.  Section 12.  No. |
|  | **Numbers Needed to Test (NNT) and Population Ancestry**  The guideline cites an NNT of 3,667 to prevent one case of SJS/TEN in a northern-European population. For UK pain services treating predominantly European ancestry populations, real-world NNTs may be even less favourable due to very low allele frequencies.  It would be valuable to:   - Provide further discussion on ancestry-adjusted NNTs specifically for UK practice. - Comment on whether universal testing remains proportionate in populations with extremely low carrier prevalence. - Consider whether a selective or ancestry-informed testing strategy may be more appropriate | The estimated NNT reported in Section 9.1 of the guideline refers to the *HLA-A*31:01* testing as assessed in a health economics study performed in the UK. Please note that the results indicated that testing for *HLA-A*31:01* is cost-effective for the National Health Services in the UK. Considering the continued reduction in genotyping costs and the feasibility of using gene-panel testing to implement this strategy, we hypothesize that pre-treatment genotyping in all patients remains proportionate. The UK CERSI-PGx does not favour a selective or ancestry-informed strategy since patients may be not aware or fail to disclose their ancestry. | No. |
|  | **NHS Laboratory Capacity and Clinical Pathways**  The guideline rightly recommends a ≤5-day turnaround for HLA typing, but no assessment of national laboratory capacity is provided. Pain services—particularly those managing severe trigeminal neuralgia—are concerned about:   - 5–7 day delays in initiating carbamazepine due to testing. - Regional variability in HLA-typing capability across the four UK nations. - Operational challenges integrating genetic testing into diverse outpatient pain pathways.   A section detailing current capacity, expected demand, and realistic implementation pathways for pain services would enhance utility and facilitate adoption. | The implementation strategy including laboratory capacity and demand in the NHS is beyond the remit of this guideline. In this regard, we expect that the publication of the guideline may be a positive driver towards implementation.  The UK CERSI-PGx guideline has been developed with the contribution of expert clinicians in the management of trigeminal neuralgia and is intended to avoid major disruptions in the current routine treatment pathway. Therefore, in Section 5.5 we recommend for all indications, if due to clinical urgency, that treatment with carbamazepine, oxcarbazepine or eslicarbazepine is started before a test result is obtained. However, we highlighted that there is still benefit in testing at the time of treatment initiation since the onset of cutaneous adverse reactions is usually delayed and the prescription can be subsequently changed. | No. |
|  | **Cost-Effectiveness and Opportunity Costs**  All economic analyses cited relate to epilepsy populations with materially different allele frequencies, clinical contexts, and treatment pathways. There is no cost-utility analysis for HLA-B*15:11, despite a recommendation to test for it.  For pain medicine, additional clarity would help:   - How cost-effectiveness conclusions translate to shorter treatment courses. - The economic impact of treatment delays in acute pain. - The opportunity cost of universal testing for low-frequency alleles in a strained NHS environment.   These considerations would support a more nuanced interpretation of the health-economic case for testing across indications. | The only published economic evaluation in the context of pain management (and HLA-B*15:02 testing) was **Rattanavipapong W at al., 2013 doi: 10.1111/epi.12325** which related to neuropathic pain in Thailand. This analysis did not consider differential costs or efficacy of alternative pain management strategies and related to a healthcare system that is substantially different from the NHS. For these reasons, and in the absence of other evidence, we consider is inappropriate to speculate on the potential impact of testing on the points highlighted.  In Section 12, we highlighted the need for additional research on *HLA-B*15:11* with respect to clinical utility of the pharmacogenetic testing and the health economics evidence. Recommendation for testing was based on the severity of the reaction that can be prevented by the adoption of the test and the increased risk observed in *HLA-B*15:11* positive patients. This is in line with recommendations from the Dutch Pharmacogenetics Working Group (DPWG) in their recent guideline (**Manson LEN et al., 2024 doi: 10.1038/s41431-024-01572-4**). | No. |
|  | **Implications for Trigeminal Neuralgia**  Carbamazepine remains a first-line therapy where alternative options are limited and often less effective. The guideline might benefit from:   - A dedicated subsection addressing testing in urgent-onset, high-severity conditions such as trigeminal neuralgia. - Specific guidance on immediate versus delayed initiation of therapy while awaiting genetic results. | We have included a specific section (Section 5.5) for treatment initiation under urgent circumstances, which applies to all indications. We have also included in section 5.3 some specific advice for trigeminal neuralgia “due to a lack of licensed alternatives that are recommended for prescribing in primary care this may result in a delay in receipt of treatment to manage pain of up to 5-7 days whilst awaiting results. Based on a shared decision-making model, a patient and prescriber may collaboratively decide whether to proceed with treatment immediately or to wait for a result, considering the specific risks based on the patient’s ancestry.” | No. |
|  | **Summary**  The scientific foundation of the guideline is strong and greatly appreciated. Further development to address the issues above—particularly pain-specific evidence gaps, ancestry-adjusted utility, operational feasibility, and economic generalisability—would significantly enhance its applicability to UK pain medicine. I welcome continued dialogue on how pharmacogenomic prescribing can best be integrated into multidisciplinary pain pathways and would be pleased to contribute to future iterations or implementation planning. | Thank you for your support. |  |
| **All Wales Therapeutics and Toxicology Centre** | No response received |  |  |
| **British Association of Dermatologists** | There are concerns about the feasibility of implementation, as many clinicians do not have access to HLA testing.  In order to improve understanding and access, it would be important to include pharmacogenetic HLA testing into the UK Genomic Test Directory, which clinicians increasingly recognise as the reference for genetic testing.  There are concerns about the full scope of cost-effectiveness. Though there is reference to health economic evaluations in epilepsy (section 9.1) and acknowledgement of the need for further economic evidence in other indications (section 12.3), a more detailed impact assessment would better detail the risk of treatment with inferior therapies--for the large number of patients who would be negative for risk alleles, but were unable to access carbamazepine due to prohibitive cost or access issues with the HLA testing.  It is not clear why patients who have been on the drug for three months would benefit from testing. Most reactions and SCARs would take place withing one month  Dermatologists generally do not prescribe these medications so it would be difficult to comment on clinical pathways and actions prior to an anticonvulsant prescription, though we would be able to support education and understanding of the wider impact of SCARs due to anti-epileptic therapy for prescribing clinicians. | We understand your concern considering the current situation availability of routine testing in the UK. The logistics of the implementation and commissioning of pharmacogenetic testing is beyond the remit of this guideline. The UK CERSI-PGx focused on the clinical utility of pharmacogenetic testing and raising awareness around the increased risk of severe cutaneous ADRs in patients who carry relevant HLA gene variants. We hope that this guideline will pave the way to implementation of testing in the NHS.  The health economics section serves to summarise the available evidence on cost-effectiveness. The health (and cost) impacts of alternative medications are captured in the costs, QALYs and the resulting incremental cost-effectiveness ratios presented. This section does not aim to estimate the impact of HLA testing more broadly.  We acknowledge the most cases of SCAR occur soon after the start of treatment, but some can be delayed for up to 3 months. Hence the reason why we have used the 3-month cut-off. This is consistent with the CPIC guideline.  Thank you for your feedback. We wanted to make you aware of this development, considering the crucial role of dermatologists in the diagnosis of SCARs due to these drugs | No.  No.  No.  No. |
| **British Society of Allergy and Clinical Immunology** | No response received |  |  |
| **NHS England Genomics Unit** | This is a really comprehensive piece of work, and it is clear a lot of work has gone into it. Therefore, I’m just providing a couple of comments discussed with colleagues in the NHSE Genomics Unit that may be helpful:  It is noted there was no scientific representation on the group, so it is important to ensure this has been validated with clinical scientist colleagues as part of the consultation. HLA testing is currently available (on request) from NHSBT who may be able to comment.  The table format for recommendations could be made more straightforward – assume HLA-A*31:01 positive and HLA-B*15:02 negative, doesn’t mean no action for positive/positive as pre the line above, but this should probably be made clearer, e.g. HLA-B*15:02 positive and any HLA-A*31:01 genotype TO HLA-B*15:02 positive and HLA-A*31:01 positive genotype OR HLA-B*15:02 positive and HLA-A*31:01 negative genotype | Thank you for your positive feedback and support.  Thanks for your comment. The guideline has been sent out for consultation to different Organizations involved in genomic analyses. We have received feedback from The Association for Clinical Genomic Science and amended the text accordingly. We also received comments by the British Society of Histocompatibility and Immunogenetics (BSHI) and The Welsh Blood Service jointly.  Please note that if *HLA-B*15:02* is positive this is the main results that will drive changes in the prescription (thus regardless the *HLA-A*31:01* or the *HLA-B*15:11* status). | No.  No. |
| **British Society of Histocompatibility and Immunogenetics (BSHI) and The Welsh Blood Service** | The guidance document has a direct relevance to BSHI members who perform the HLA testing assays described. The following comments have been received after circulation to the ~20 NHS H&I laboratories.  **Section 6: Gene(s), Variants, and Turnaround Time**  **BSHI Comments - Assay Recommendations**  The guidelines state: *“We recommend direct HLA typing laboratory methods rather than the use of tag-SNPs – the latter infer HLA alleles based on linkage disequilibrium (LD) patterns, which can lead to misclassification in genetically diverse populations because of weaker LD patterns.”*  We welcome this recommendation but believe further clarification is required. The term ‘direct HLA typing method’ is a wide definition and may include assays that provide intermediate-resolution HLA types, generating a list of possible alleles that may include the risk genotype as the most likely.  For example, commercially available PCR-SSP HLA typing assays, such as Ready Gene HLA typing (inno-train Diagnostik GmbH, Germany), are direct typing assays. These can identify HLA-B*15:02 but cannot exclude rarer alleles (e.g., HLA-B*15:21, 15:44, 15:88, 15:121, and others). While these alleles are unlikely, they remain possible.  We recommend revising the guideline to reflect WHO HLA nomenclature and specify the required resolution level. Suggested wording:  *“**HLA typing should be performed to a minimum of two-field resolution for HLA-B*15:02, B*15:11, and A*31:01 alleles, and any ambiguities associated with the results should be reported.”*  Two-field resolution requires technology capable of identifying the implicated allele only. Currently, this is most commonly achieved via NGS typing, although alternative allelic-level resolution assays are available.  **BSHI Comments – Reporting Turnaround Times**  The recommended turnaround time of five days exceeds the routine turnaround reported by several BSHI member laboratories for high-resolution HLA typing. Many laboratories report 7-14 working days from receipt for routine testing, primarily due to workload prioritisation and resource constraints.  Typical CE/UKCA-marked NGS-based approaches take approximately 24 hours from start to finish. However, as most laboratories do not operate 24-hour services, assays are staggered across routine working hours, resulting in a practical duration of 48-72 hours. While urgent requests can be prioritised, achieving a five-day turnaround for routine pharmacogenomic testing is challenging under current conditions.  We suggest the guideline acknowledge that current UK turnaround times are typically 7-14 days, with prioritisation for cases of clinical urgency.  **BSHI Comments – Audit, Quality, and Competence Requirements**  To ensure reliable and accurate results, laboratories undergo independent audits. We recommend the guideline include the following statement: *“**Laboratories performing HLA genotyping for alleles associated with increased risk of severe cutaneous adverse reactions to carbamazepine and related compounds should hold ISO 15189 accreditation and/or European Federation for Immunogenetics accreditation.”*  **BSHI Comments – Minor comments**  Occasionally B*1502 instead of B*15:02 is used. | Thank you for your positive comment and support of the guideline.  Thank you for this relevant observation. The text has been amended as suggested.  Thank you for your comment. The UK CERSI-PGx is aware that current turnaround time for HLA typing is longer than what is recommended in our guidelines. However, we hope that the publication of this guidance may be a driver to provide genotyping results within the time frame (laboratory time) of less than 5 days, as this is considered the optimal timing for initiation of therapy in most clinical scenarios.  Thanks for your comment. We included your recommendation in the guideline.  The text has been checked for consistency | Section 6.  No.  Section 6.  All text. |
